# An experimental investigation into whether choice architecture interventions are considered ethical

**DOI:** 10.1038/s41598-023-44604-7

**Published:** 2023-10-26

**Authors:** Daniella Turetski, Renante Rondina, Jordan Hutchings, Bing Feng, Dilip Soman

**Affiliations:** 1 Rotman School of Management, University of Toronto, Toronto, ON Canada; 2 Treasury Board of Canada Secretariat, Ottawa, ON Canada

**Keywords:** Human behaviour, Psychology

## Abstract

**Abstract:**

Despite their increasing use, choice architecture interventions have faced criticism for being possibly manipulative and unethical. We empirically explore how an intervention’s acceptability differs by the type of intervention used, by the domain, and by the way in which its implementation and benefits are explained. We employ a 5 × 5 × 5 factorial design with three fully crossed predictor variables: domain, type of intervention, and explanation. We measure participants’ acceptance of the proposed intervention, perceived threat to autonomy and freedom of choice, and belief that the intervention will be successful. We hypothesized that acceptability of the intervention and perceived threat to autonomy will change as a function of the type of intervention used, the domain in which it is implemented, and the rationale for which its use is presented. We find that acceptability of the intervention, perceived threat to autonomy, and belief that the intervention will be successful differ by the type of intervention used and by the domain in which it is implemented. The rationale for the use of the intervention appears to change acceptability of the intervention depending on the type of intervention that is being used, and the domain in which it is implemented. Exploratory analyses were conducted to investigate differences between specific levels within factors, and interactions between factors. Given the variation in acceptability across the three factors, we believe that the discourse about the ethics of choice architecture should avoid generalizations and should instead be at the level of individual interventions in a specific situation. We conclude with a discussion about areas for future research.

**Protocol registration:**

The stage 1 protocol for this Registered Report was accepted in principle on 14 October 2022. The protocol, as accepted by the journal, can be found at: 10.6084/m9.figshare.21758666.

## Introduction

Choice architecture—“organizing the context in which people make decisions”^1^—has gained immense popularity in applied behavioural science. Many governments and public sector organizations have used choice architecture to help individuals make better choices for themselves^2^. Private sector organizations also use similar strategies to influence customers^3^, often to advance the organization’s own commercial objectives, but also to achieve progress towards socially beneficial goals, such as reduced residential energy usage^4^. In principle, this approach is thought to preserve an individual’s autonomy and freedom of choice, relative to bans and mandates, and has generally been accepted by the public in many countries^5,6^. However, choice architecture has also received criticism for being possibly manipulative and insidious (e.g., perceived threat to the target’s autonomy, the intervention lacking transparency)^7–11^.

Much has been written in the scientific space about ethical issues surrounding choice architecture (or nudging, as it is popularly called)^12–15^. However, for a field that is heavily experimental in nature, little experimental work has been done to investigate the perceived ethics (e.g., acceptability and perceived threat to autonomy) of choice architecture interventions. While there is suggestive evidence of reactance against certain types of interventions^16^, we wish to experimentally explore how reactions differ across various scenarios. In particular, how do the ethics of an intervention vary across specific interventions, the domains in which they are delivered, and how the rationale for their use is presented?

### Ethics as a function of intervention type

There are a variety of choice architecture interventions that have been used, ranging from defaults to reminders. One important facet of these interventions is their perceived level of intrusiveness. Prior research suggests that the acceptability of an intervention and its level of intrusiveness have an inverse relationship^17^. Interventions have also been distinguished based on whether they are “nudge” interventions (use choice architecture to steer people) or “boost” interventions (empower people to overcome context effects, typically through education, decision support or interventions that highlight the relevance of information)^18^. Experimental evidence shows that people tend to find “boosts” more acceptable than “nudges”^19,20^. Another stream of literature supporting this finding explores the acceptability and perceived threat of autonomy of default options (a type of “nudge”). Default options tend to be particularly threatening to one’s sense of autonomy^14,21–25^. This research, especially the work done by Michaelsen and coauthors (2021), has begun to empirically explore the perceived acceptability of specific interventions (i.e., default options). Their findings suggest that acceptability may vary across different types of interventions. Our aim is to investigate the perceived ethics of choice architecture interventions across a broader range of interventions, rather than focusing solely on default options.

#### H1

(a) Acceptability ratings and (b) perceived threat to autonomy will vary depending on the *type of intervention* used (i.e., defaults, incentives, salience, reminders, social proof).

### Ethics as a function of domain

Interventions differ in terms of the behaviour they are targeting and the intended impact of the behaviour change (i.e., the domain in which the intervention is implemented). For example, default options have been used to increase vaccination rates^26^, as well as retirement savings plan contributions^27^, and social norm messages have been used to improve pro-environmental behaviours^28,29^ and healthy eating behaviours^30^. It has been suggested that choice architecture interventions may be more effective and appropriate in some domains over others^31^. For example, while it seems that public opinion tends to agree more on the ends of an intervention than its means^15^, some studies have found differences in opinions depending on who benefits from the intervention (which changes across different domains). People find interventions that are intended to benefit the individuals being influenced to be more acceptable and less autonomy-threatening than interventions that are intended to benefit society in general^32–34^. Interventions also differ in terms of the agent doing the nudging (e.g., public sector versus private sector organizations). However, to the best of our knowledge, there are no studies comparing the perceived ethics of interventions delivered by different agents. These findings suggest that the domain in which the choice architecture intervention is implemented may affect its perceived ethics, and we aim to explore whether this is the case.

#### H2

(a) Acceptability ratings and (b) perceived threat to autonomy will vary depending on the *domain* in which the intervention is implemented (i.e., organ donation, retirement savings, flu shots, flood insurance, electric vehicles).

### Ethics as a function of rationale

Perceived ethics may also depend on the rationale provided on the use of an intervention in any given domain. For example, in the domains of retirement savings and carbon emission offsets, people tend to have more favorable opinions about the use of default options when the ability to resist the choice architecture at low cost is highlighted, but not with organ donations^34^. In the domain of food-related interventions, highlighting the effectiveness and resistibility of the choice architecture were positively correlated with greater acceptability of the intervention^35^. Similar to resistibility, we believe that emphasizing that one can choose not to engage with the intervention (e.g., can opt-out of the default option) may impact the perceived ethics. Opinions may also be influenced if the rationale is framed in terms of the losses that the intervention is intending to prevent. *Loss aversion*^36^ can explain why penalty frames are sometimes more effective than reward frames in motivating people^37^. This suggests that framing the consequences of not using an intervention in a negative way (i.e., as a loss) could improve people’s perceptions of it. Therefore, resistibility, emphasizing one’s ability to choose, effectiveness, and loss framing represent examples of different rationales for explaining the use of an intervention that may potentially affect public opinions on the ethics of choice architecture interventions.

#### H3

Acceptability ratings and (b) perceived threat to autonomy will vary depending on the *rationale* used to explain the intervention’s implementation and benefits (i.e., control, effectiveness, choice, loss aversion, resistibility).

### The present research

The aforementioned literature suggests that the ethics of an intervention might depend on the type of intervention, the domain, and the rationale provided for it. However, to our knowledge, the effects of each of these factors have never been simultaneously manipulated and experimentally documented. In this registered report, we presented participants with hypothetical scenarios in which different types of choice architecture interventions were planned to be delivered across different domains. We provided different rationales for the use of each intervention and asked participants to rate each intervention in terms of its acceptability, perceived threat to autonomy or freedom of choice, and potential for success. The three factors were fully crossed so that we could determine the effect of each factor, and explore any potential interactions between factors. Intuitively, since we manipulated the rationale of the intervention, we expected that this would shift perceptions of potential for success. Indeed, the literature shows that acceptance of a choice architecture intervention is positively related to its perceived effectiveness^38^. However, we had no specific hypotheses about how perceived effectiveness would be affected by the type of intervention or the domain. We thus included a measure of perceived potential for success to test our expectation that manipulating rationale should affect perceived potential for success, and as an exploratory measure.

## Methods

### Ethics information

The experiment was approved by the Research Ethics Board at the University of Toronto.

Participants were compensated $6US for completing the experiment.

### Pilot data

This registered report was based on a pilot experiment (see Supplementary Information 1) that originally employed a Latin Squares design with three factors that had five levels each: Domain (the context in which an intervention was employed), Type (the nature of the intervention—referred to as *Intervention* for the main registered report), and Rationale (how the rationale for the intervention was framed). We collected data from 150 participants who responded to five scenarios such that each participant was exposed to each level of each factor exactly once. We asked participants to rate the acceptability, perceived threat to autonomy, and perceived potential for success of the intervention employed in each scenario. For ratings of acceptability, we found an effect of Domain (*F*_(4,583.02)_ = 3.15, *p* = 0.0140, partial eta-squared = 0.02), an effect of Type (*F*_(4,583.02)_ = 10.78, *p* < 0.0001, partial eta-squared = 0.07), and an effect of Rationale (*F*_(4,583.02)_ = 5.93, *p* = 0.0001, partial eta-squared = 0.04). For ratings of autonomy, we found an effect of Domain (*F*_(4,583.18)_ = 2.52, *p* = 0.0402, partial eta-squared = 0.02), an effect of Type (*F*_(4,583.18)_ = 11.35, *p* < 0.0001, partial eta-squared = 0.07), an effect of Rationale (*F*_(4,583.18)_ = 2.67, *p* < 0.05, partial eta-squared = 0.02), and a main effect of Group (*F*_(4,583.18)_ = 2.45, *p* = 0.031, partial eta-squared = 0.06). For ratings of success, we found an effect of Domain (*F*_(4,583.23)_ = 3.10, *p* = 0.0152, partial eta-squared = 0.02), and an effect of Type (*F*_(4,583.23)_ = 5.49, *p* = 0.0002, partial eta-squared = 0.04).

### Design

The experiment was built and hosted in Qualtrics. It was run online and was made compatible with cellular devices and tablets. Participants had no contact with researchers before or during the experiment, so both participant and researchers were blind to group assignment. We informed each participant that they would have to read six scenarios in which an organization or government was attempting to nudge their customers or citizens (five scenarios from a possible 125, and one extreme case as a baseline condition). Participants were told that a “nudge” is a way of presenting choices and information in such a way to encourage people to select the option or behave in such a way that promotes their best interests or general welfare. Participants were also informed that after each scenario, they would be asked to indicate how much they agreed or disagreed with the intervention (*acceptability*), how much it limited their freedom of choice (*perceived threat to autonomy*), and how effective they think the intervention would be.

All stimuli were displayed in dark grey text against a white background (we preregistered that the text would be pure black, but the default dark grey on Qualtrics was more natural). Each scenario was structured as three paragraphs, with a line and no headings between paragraphs. The first paragraph introduced the organization or government, the behaviour they were trying to change, and the positive impact it would be expected to have on the customers or citizens. The second paragraph described the status quo and the changes that were planned to be made. The third paragraph consisted of one short sentence that either reiterated the benefits or effectiveness of the intervention, the risk of not complying with the target behaviour, or the customer or citizen’s ability to resist or exempt themselves from the planned changes. At the bottom of each scenario, participants were asked to indicate how much they agreed or disagreed with the following statements:

- I find the proposed changes acceptable.
- I approve of the proposed changes.
- I agree with the way in which the change will be presented.
- The proposed changes do not threaten my autonomy or freedom of choice.
- The way in which the change was presented to me tried to manipulate me.
- The way in which the change was presented to me tried to make decisions for me.
- I believe the proposed changes will successfully produce the intended effects.

Participants were instructed to indicate their response on a 7-point Likert scale (1 = strongly disagree, 4 = neither agree nor disagree, 7 = strongly agree). These measures were adapted from Sunstein (2016), as well as the Dillard and Shen (2005) threat to autonomy items used in Michaelsen et al.^16,24,39^.

We employed a 5 × 5x5 factorial design with Domain, Intervention, and Rationale as fully crossed predictor variables (see Supplementary Information 2 and 3). For Domain, the first paragraph introduced the scenario by describing a government or organization, the behaviour of their citizens or customers that they were trying to change, and the intended positive outcomes of the behaviour change (Organ Donation, Retirement Savings, Flu Shots, Flood Insurance, Electric Vehicles). For Intervention, the second paragraph described the status quo and the type of intervention being used (Defaults, Incentives, Salience, Reminders, Social Proof). For Rationale, the third paragraph contained a short sentence to test different ways that a participant’s ratings may change by altering the way in which an intervention’s benefits and implementation were explained (Control, Effectiveness, Choice, Loss Aversion, Resistibility). In the control condition, no rationale of the intervention was provided. Rather, the intended behaviour change was reiterated. Five versions of the second and third paragraphs were created for each level of Intervention and Rationale so that they were compatible with each level of Domain. This produced 125 unique scenarios that were randomly and evenly divided across 25 groups. These interventions, domains, and rationales were chosen to cover as diverse of a range of scenarios as possible and to feature the most popular interventions and domains. Participants were randomly and evenly assigned to one of the 25 groups such that participants in the same group viewed the same 5 unique scenarios, but each group viewed a different set of 5 scenarios. This way, all versions of all scenarios were covered between participants in the 25 groups, but an individual participant only viewed 5 of the 125 possible scenarios (as well as a sixth baseline scenario that was common to all participants).

At the start of the experiment, as an attention check, participants were told a non-sensical piece of information which they needed to answer a question about. If they did not answer correctly, they did not continue with the study. After responding to the five scenarios (plus the sixth baseline scenario), participants were also asked to respond to five quality assurance questions that served as an additional awareness check. Each question cued the participant with one of the five domains from the experiment (not the baseline scenario) and asked the participant to recall the intervention that was employed in that domain. For each question, participants were instructed to make their responses by selecting one of the five interventions. These attention checks together aided in filtering out both bots and participants who may have been quickly and aimlessly answering the questions.

### Sampling plan

We aimed to recruit 22 participants per group. An a priori power analysis was conducted using G*Power version 3.1.9.7 for sample size estimation^40^, based on our pilot data. An effect size of 0.204 was calculated using a partial eta-squared of 0.04. This was the partial eta-squared of the main effect of Rationale from our pilot data analyzing acceptability ratings (we chose the smallest effect size to be conservative; see Supplementary Information 1). With a significance criterion of α = 0.05 and power = 0.95, the minimum sample size needed with this effect size was *N* = 451 for our analyses. Therefore, 22 participants per group for 25 groups would amount to *N* = 550 (over-recruiting by 22%) which would be sufficient to test our hypotheses, even with anticipated failures and participants exclusions. Participants were adults (18+ years) from the United States or Canada who signed up for the study through Amazon’s Mechanical Turk. We excluded and replaced any participant that answered more than two quality assurance questions incorrectly, any participants who did not pass our non-sensical question attention check, as well as any participants that did not finish all the questions (so that we had five complete data points per participant).

### Analysis plan

Data were exported from Qualtrics and restructured for analysis in R using the dplyr and tidyr packages^41,42^. We originally preregistered that we would restructure the data using the reshape2 package, but later realized that the dplyr and tidyr packages were a cleaner and more efficient strategy, without impacting any of our preregistered analyses. All analyses were conducted using the ordinal^43^ and RVAideMemoire^44^ packages. Scores of acceptability, autonomy, and success were each fitted into a separate cumulative link mixed model (CLMM) with Domain, Intervention, and Rationale as fixed factors and Subject as a random intercept and analyzed with a type 3 ANOVA. We preregistered that we would average ratings for the three statements measuring acceptability and three statements measuring threat to autonomy to create acceptability and autonomy scores. However, averaged scores pose a problem for CLMM models. Instead, we switched to including participant ID as a random effect for each of the two CLMM models, as per the recommendation from the editorial team. As an exploratory analysis, we repeated the analysis again but with all possible interactions. We used an alpha level of 0.05 for all our analyses. We also conducted a variety of post hoc analyses to explore differences between specific levels within factors (see Supplementary Information 1 for examples from our pilot data and see the results section for an explanation of why we do not Bonferroni correct for multiple comparisons as initially preregistered). For the registered report, we reran similar post-hoc analyses, as well as additional ones. We also took advantage of our fully crossed design to explore interactions between factors, though we did not have any specific hypotheses about them.

## Results

### Sample recruitment

We recruited our first batch of 550 participants, as per the registered protocol. Informed consent was obtained from all participants, and this study was conducted in accordance with the ethical guidelines of the University of Toronto. Of these participants, 41% passed our quality assurance attention checks. This was greater attrition than we had originally accounted for. To attain our preregistered sample of at least 451 participants, we recruited an additional batch of 590 participants, and then a third batch of 105 participants. In this third batch, we targeted two specific groups (of the 25 groups) that had particularly high attrition rates, so that we could ensure that enough participants were in each group to do any interaction and within-factor exploratory analyses (see Supplementary Information 4 for participant numbers per group after exclusions). To ensure that the date on which we recruited participants does not affect our results, we included batch date as a factor in our analyses. It only had an effect in the Autonomy model (*X*^2^ (2, *N* = 483) = 11.245, *p* = 0.004 (all *p* values in other models fall above 0.25)). However, upon further examination, this effect seems to be a result of the third, final batch in which we targeted two specific groups, and not because of the batch date itself. To further ensure that the recruitment of additional participants did not introduce any bias into our sample, we reran all our main analyses using just the initially recruited batch of participants. We did not observe any changes in the directions of our main effects (except for the effect of Domain in the Success model; see Supplementary Information 5). After all exclusions, we were left with a final sample of *N* = 483.

We preregistered that we would collect demographic information (age, sex, and country of residence). Unfortunately, in the process of converting the pilot design into the present study, the section for demographic data was not included. This oversight was only noticed after data collection had been completed. As we cannot report the representativeness of our sample, please keep this in mind when discussing the generalizability of our results. However, through Mechanical Turk, we did input that we want to recruit evenly across genders, and from the United States and Canada only (all participants were 18+).

As an additional layer for ensuring that our data are of good quality, we included a sixth baseline condition that was common to all participants in all groups. The purpose of this condition, as preregistered, was to serve as an extreme scenario that we expected would catch the attention of most participants, thus helping us understand whether participants were aimlessly clicking through our questions, or whether they were thinking through their answers thoroughly (as we expected, from prior literature^16^, that this scenario would be rated more negatively than our other scenarios). We found that participants’ responses to the acceptability and autonomy questions for the five non-baseline scenarios were significantly different, on average, from the baseline condition (see Appendix 1). Thus, the results from the acceptability and autonomy responses make us confident that the participants in our post-exclusions sample thought through their responses carefully and responded thoughtfully. Overall, given these results and our stringent attention checks, we have ensured that the quality of our dependent variables is sufficient to detect real differences in our data and thus test our research questions.

### Main effects analyses and exploratory interaction analyses

As preregistered, we fit scores of acceptability, autonomy, and success into separate cumulative link mixed models (CLMM) with Domain, Intervention, and Rationale as fixed factors. We analyzed our hypothesized main effects (H1–H3) and explored interactions using a type 3 ANOVA. Two of the threat to autonomy items were reverse-coded in the analyses such that a higher score means the intervention is perceived as less autonomy-threatening.

#### Acceptability

We hypothesized that an intervention’s acceptability would differ by domain, the type of intervention used, and the way in which its implementation and benefits are explained (H1a, H2a, H3a). In our pilot data, we found an effect of all three of these factors. In this study, however, we only observe significant effects of domain and the type of intervention used (see Table 1). We also observe a significant Intervention*Domain interaction, suggesting that certain types of interventions may differ in acceptability depending on their domain, or that certain domains may be more acceptable for implementing choice architecture interventions depending on what type of intervention is used. While we do not observe our hypothesized main effect of rationale, upon further analysis of the interaction terms, we find that the Intervention*Rationale interaction is significant and the Domain*Rationale interaction is marginally significant, suggesting that the way in which an intervention’s implementation and benefits are described might matter depending on the specific intervention and domain. We explore this further in the exploratory within-factor analyses.

**Table 1:** The main effects of domain, intervention, and rationale, as well as all 2-way and 3-way interactions.

	Acceptability			Autonomy			Success		
	ChiSq	Df	*p*	ChiSq	Df	*P*	ChiSq	Df	*p*
Domain	25.723	4	< 0.001***	23.659	4	< 0.001***	12.782	4	0.012**
Intervention	283.411	4	< 0.001***	195.679	4	< 0.001***	24.865	4	< 0.001***
Rationale	6.690	4	0.153	3.579	4	0.466	2.035	4	0.729
Domain*intervention	33.671	16	0.006***	15.955	16	0.456	19.241	16	0.256
Domain*rationale	23.483	16	0.101	8.166	16	0.944	8.425	16	0.935
Intervention*rationale	31.580	16	0.011**	14.567	16	0.557	10.605	16	0.833
Domain*intervention*rationale	68.694	64	0.321	39.723	64	0.993	40.448	64	0.991
Batch date	2.766	2	0.251	11.245	2	0.004***	1.478	2	0.478
Counterbalance group	− 0.116	24	1	− 0.225	24	1	− 0.006	24	1

We include counterbalance group and batch date as factors to check for any effects of group assignment and date of batch collection.

*p<0.1; **p<0.05; ***p<0.01.

#### Autonomy

As with acceptability, we hypothesized that an intervention’s perceived threat to autonomy would be affected by the domain, the type of intervention used, and the rationale to explain its benefits and implementation (H1b, H2b, H3b). While in our pilot data we found that all three of these factors were significant, here we only observe that the type of intervention and domain have a significant effect on perceived threat to autonomy (see Table 1). We do not observe our hypothesized main effect of rationale, and we do not observe any significant interactions.

#### Success

Participants’ beliefs for how successful the intervention will be were included mainly as an exploratory measure, but also as a check for our expectation that our rationale manipulations should affect this measure. We observe that both the domain and the type of intervention used have a significant effect on the belief that the intervention will be successful (see Table 1). Counter to our intuition, the way in which the intervention was described (rationale) does not have a significant effect on belief that the intervention will be successful. We do not observe any significant interactions.

### Exploratory within-factor analyses

**Figure 1:**
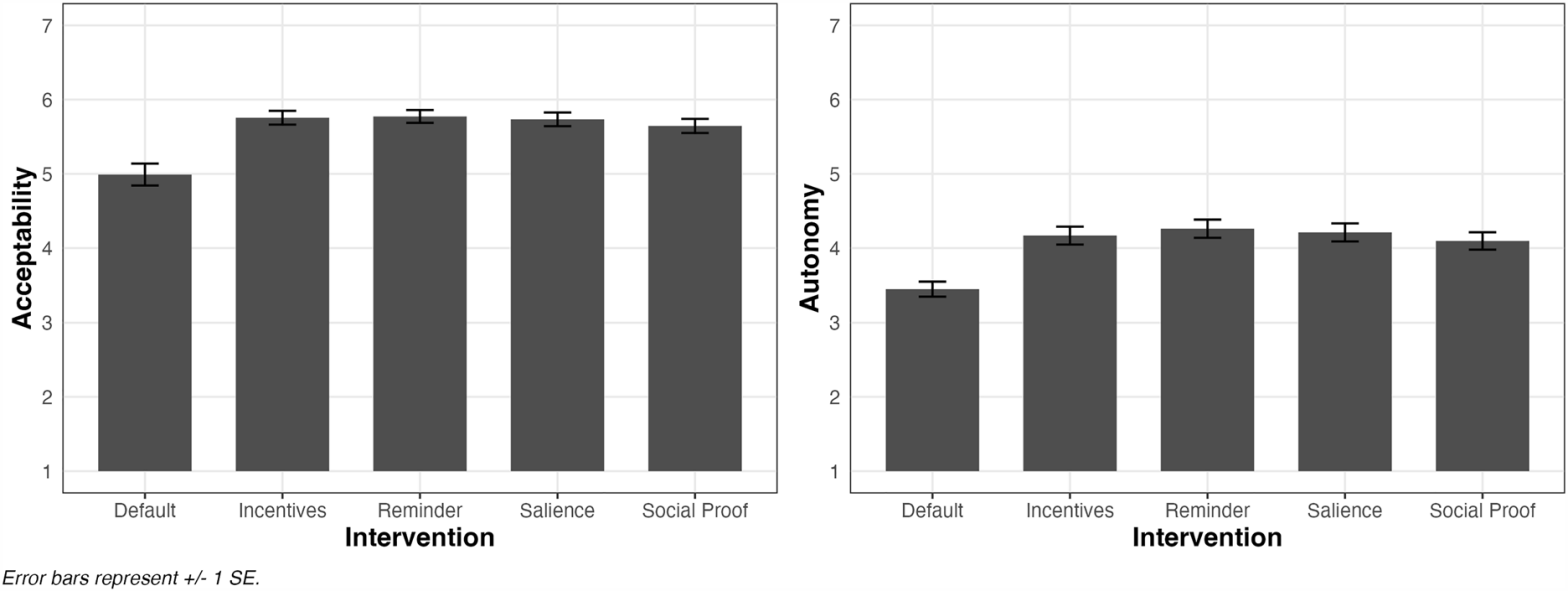
**Average ratings of acceptability and perceived threat to autonomy for each level of Intervention**. Across all of our dependent variables, higher scores are interpreted as the intervention being perceived as more ethical. For acceptability, higher scores mean the intervention is rated as more acceptable. For autonomy, higher scores mean the intervention is less autonomy-threatening.

Across the different types of interventions, different domains, and different rationales, the only factor with significant differences between levels was Intervention. Defaults were, on average, rated as significantly less acceptable and more autonomy-threatening than all the other interventions (see Fig. 1). Past research finds an inverse relationship between the level of intrusiveness of an intervention and its acceptability^17^. Defaults may be on the higher end of what is considered intrusive, at least in comparison to the other interventions in our study. This may have contributed to their lower acceptability and higher perceived threat to autonomy.

To better identify the pattern of data within one factor while holding the others constant, we plotted average acceptability, autonomy, and success scores in three plots (see Appendix 2).

We avoided making any causal claims in the within-factor comparisons and opted to descriptively highlight possible areas for future research. In order to prevent false conclusions being drawn from these descriptive findings, we chose to not adjust our confidence intervals to account for multiple hypothesis testing (i.e., Bonferroni corrected standard errors as initially preregistered). We are simply highlighting some instances in which it appears as though there is an interesting pattern, by visually exploring the plots with 95% confidence intervals. Given the sheer number of comparisons, the likelihood of some differences appearing simply by chance is high. We, therefore, avoid making any definitive conclusions and instead opt to highlight some patterns in our data. This decision was made before we conducted any of our analyses, as we decided that our study was underpowered for the number of pairwise comparisons that are possible (we present the plot with Bonferroni corrected confidence intervals in Supplementary Information 6).

#### Rationale

Given the significant Intervention*Rationale interaction and marginally significant Domain*Rationale interaction in the acceptability model, we first explore differences within the rationale factor. In these cases, the domain and type of intervention are held constant, and we explore how differences in the intervention’s rationale appear to affect its acceptability. Prior research in the domain of food interventions has shown a positive relationship between highlighting an intervention’s effectiveness and its acceptability. However, our exploratory analyses show that highlighting effectiveness does not appear to be a universally positive strategy. For incentives in the domain of flood insurance, highlighting effectiveness appears to improve acceptability relative to loss aversion framing. For incentives in the domain of flu shots, highlighting effectiveness appears to improve acceptability relative to emphasizing one’s ability to choose to opt out. However, for incentives in the domain of retirement savings, highlighting effectiveness appears to be not as good as emphasizing one’s ability to resist the intervention. We observe this same pattern of effectiveness appearing to negatively impact acceptability relative to resistibility in the domain of retirement savings for salience interventions as well.

#### Intervention

In our data, we found that defaults were, on average, rated as significantly less acceptable and more autonomy-threatening than all the other interventions. Does this lower acceptability of defaults vary across different domains and in scenarios where different rationales are used? Our data suggest that defaults are universally rated as less acceptable than other interventions. In fact, it is hard to find situations where defaults are not amongst the most unacceptable interventions in our data.

There were also a few situations in which social proof interventions are rated as less acceptable than other interventions. For example, social proof interventions that use a loss framing rationale in the domain of organ donations appear to be less acceptable than reminder interventions using the same rationale in the same domain. Similarly, social proof interventions that emphasize one’s ability to resist in the domain of organ donations appear to be less acceptable than reminder interventions in the same domain and using the same rationale.

#### Domain

Holding intervention type and rationale fixed, we observe that the acceptability of choice architecture interventions varies across domains. For example, salience interventions that emphasize effectiveness appear to be less acceptable in the domain of retirement savings compared to the domain of flu shots. We find a similar pattern for incentives that emphasize effectiveness with retirement savings appearing to be a less acceptable domain relative to flu shots and flood insurance. However, other retirement savings interventions appear to be rated as relatively more acceptable. For example, reminders that use loss framing appear to be more acceptable in the domain of retirement savings than electric vehicles, and incentives that emphasize resistibility appear to be more acceptable in retirement savings relative to organ donation.

## Discussion

We originally hypothesized that an intervention’s a) acceptability ratings and b) perceived threat to autonomy would vary based on the type of intervention used (H1a, H1b), the domain in which it is implemented (H2a, H2b), and the rationale used to explain its implementation and benefits (H3a, H3b). We find evidence to support H1a, H1b, H2a, and H2b. We do not observe a main effect of rationale on acceptability and threat to autonomy ratings (H3a, H3b). Evidently, acceptability ratings and perceived threat to autonomy ratings depend on the type of intervention used as well as the specific domain in which the intervention is being implemented. We also explored beliefs about the intervention’s success, mostly to test our intuition that manipulating rationale should subsequently affect success ratings. Interestingly, we find an effect of intervention type and domain on anticipated success, but do not observe an effect of rationale on success ratings. Individuals’ beliefs about the success of an intervention thus depend both on the type of intervention being discussed as well as the domain in which the intervention is being implemented.

Upon further analysis of the interaction terms in the acceptability and autonomy models, we observed a significant Intervention*Rationale interaction and marginally significant Domain*Rationale interaction in the acceptability ratings model. Through various additional exploratory between- and within-factor analyses, we investigated how rationale interplays with domain and intervention type to affect acceptability ratings. In doing so, we hope that we have demonstrated that acceptability of choice architecture interventions is highly varied as a function of the specific intervention and the context in which it is deployed.

Through this registered report, our aim was to explore the effects of intervention type, domain, and provided rationale on an intervention’s perceived acceptability and threat to autonomy—two aspects of perceived ethics. While this result might seem intuitive and unsurprising, we believe it is important for it to be tested and documented in an empirical way. To the best of our knowledge, we are the first to simultaneously experimentally manipulate each of these factors to tease apart how ethical the intervention is perceived to be. While our goals in this report were modest, we believe that some of our results are worthy of further exploration. For example, future research could better tease apart what drives acceptability of different interventions in different domains when the intervention’s effectiveness is emphasized as a rationale for its implementation. Future research should also continue to explore why defaults are systematically rated as less acceptable and more autonomy-threatening than other interventions, and how acceptability of other interventions (e.g., social proof interventions) differs across different domains and rationales. Future research is also required to understand which unique aspects of different domains cause choice architecture interventions to be perceived as more vs. less acceptable. Perhaps some interventions are more acceptable in private (e.g., electric vehicles) vs. public (e.g., organ donation) domains. Or some domains more directly affect a specific individual (e.g., retirement savings) vs. the greater population (e.g., flu shots). There are also a variety of subgroups that future research could explore. For example, even when most individuals accept an intervention, there may be small subgroups with very extreme negative reactions that greatly impact the overall results of studies testing perceived ethics. Future research could examine patterns of responses towards the use of choice architecture interventions amongst these different subgroups, as well as qualitative differences in attitudes across subgroups using methods such as text analysis.

In sum, our data allow us to make the following key points:

1. It is important to study the ethics of choice architecture interventions empirically.
2. We should avoid making generalized statements about the ethics of choice architecture interventions, and instead focus on exploring specific implementations of choice architecture interventions to better understand their acceptability in the public eye.
3. The encouraging news is that individuals (in our study) tended to find most of the scenarios acceptable and not-threatening to autonomy.
4. More research is needed, but we need to move the discourse beyond simply asking, “are choice architecture interventions ethical?”

## Supplementary Information

Appendix.

Supplementary Information.

Figure Legend.

## Data Availability

The data and materials for this study have been made available at: https://github.com/jjhutchi/CAEthics.

## Code availability

The R code for analysis for this study has been made available at: https://github.com/jjhutchi/CAEthics.
