# Supplementary material for: An experimental investigation into whether choice architecture interventions are considered ethical
Source: Sci Rep. 2023 Oct 26;13:18334. doi: 10.1038/s41598-023-44604-7 (PMC10603073; doi:10.1038/s41598-023-44604-7)
Supplement: Supplementary file 1 — Appendix. [file 41598_2023_44604_MOESM1_ESM.pdf]

Appendix 1. Baseline Condition Results

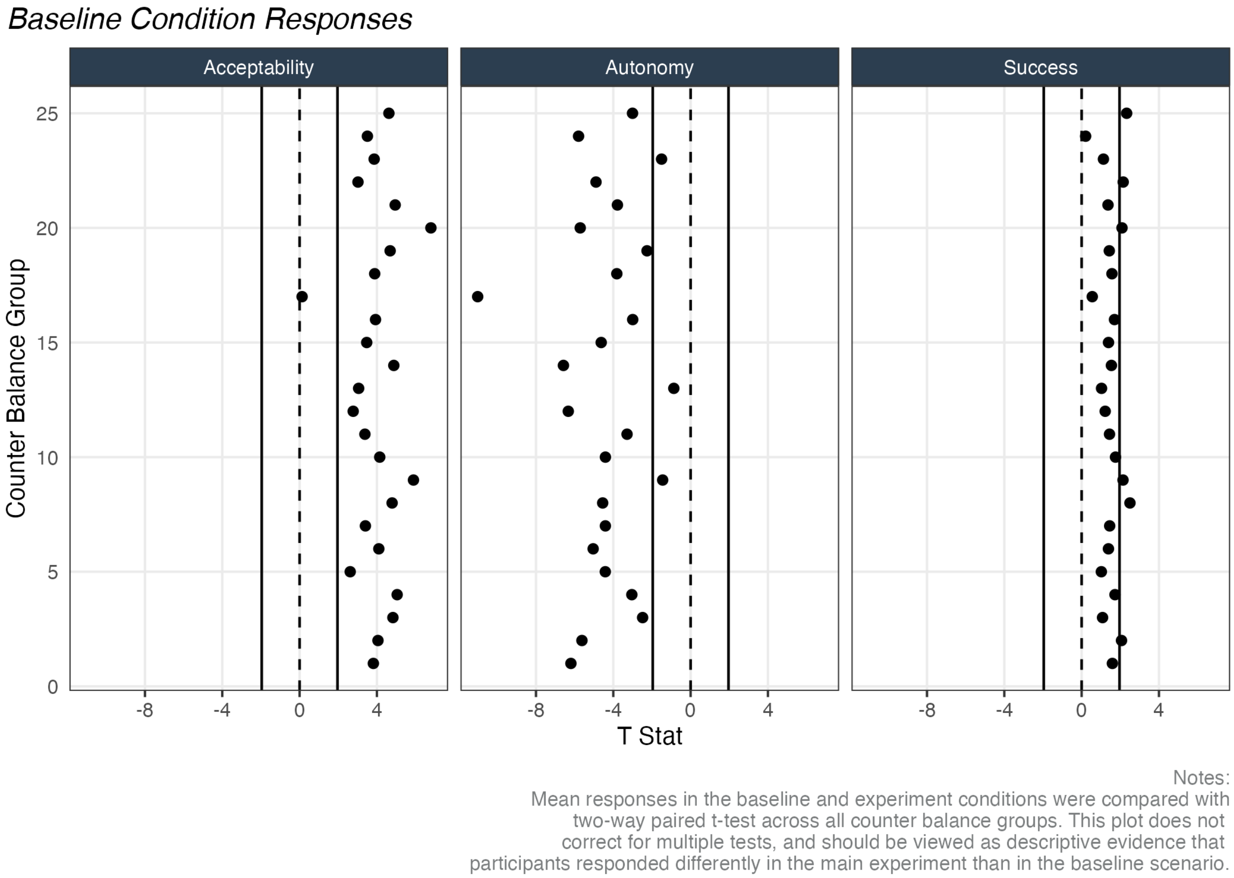

Appendix 2. Plots of differences within one factor holding the other two constant.

2.1 Exploring differences between levels of Rationale

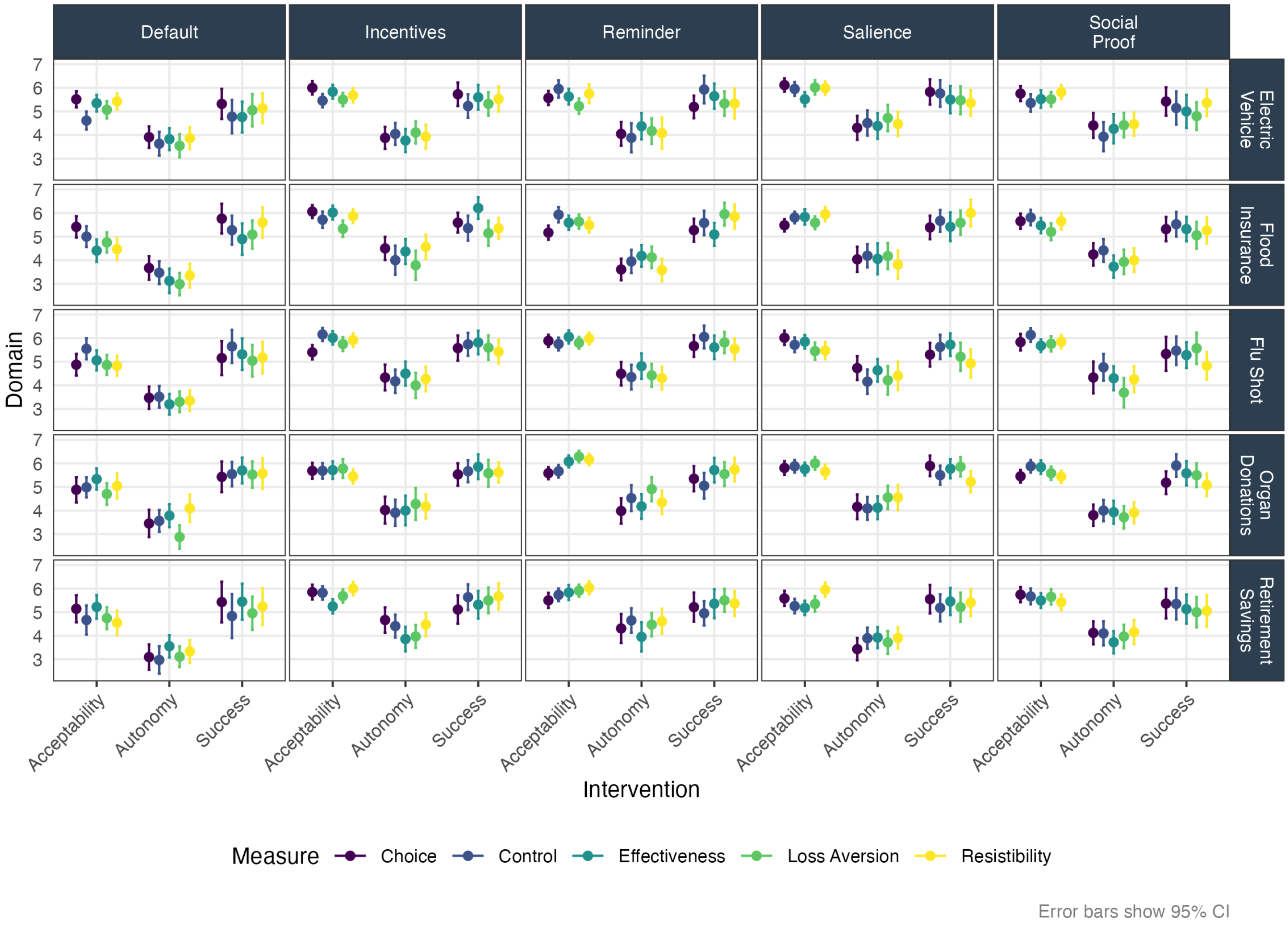

2.2 Exploring differences between levels of Intervention

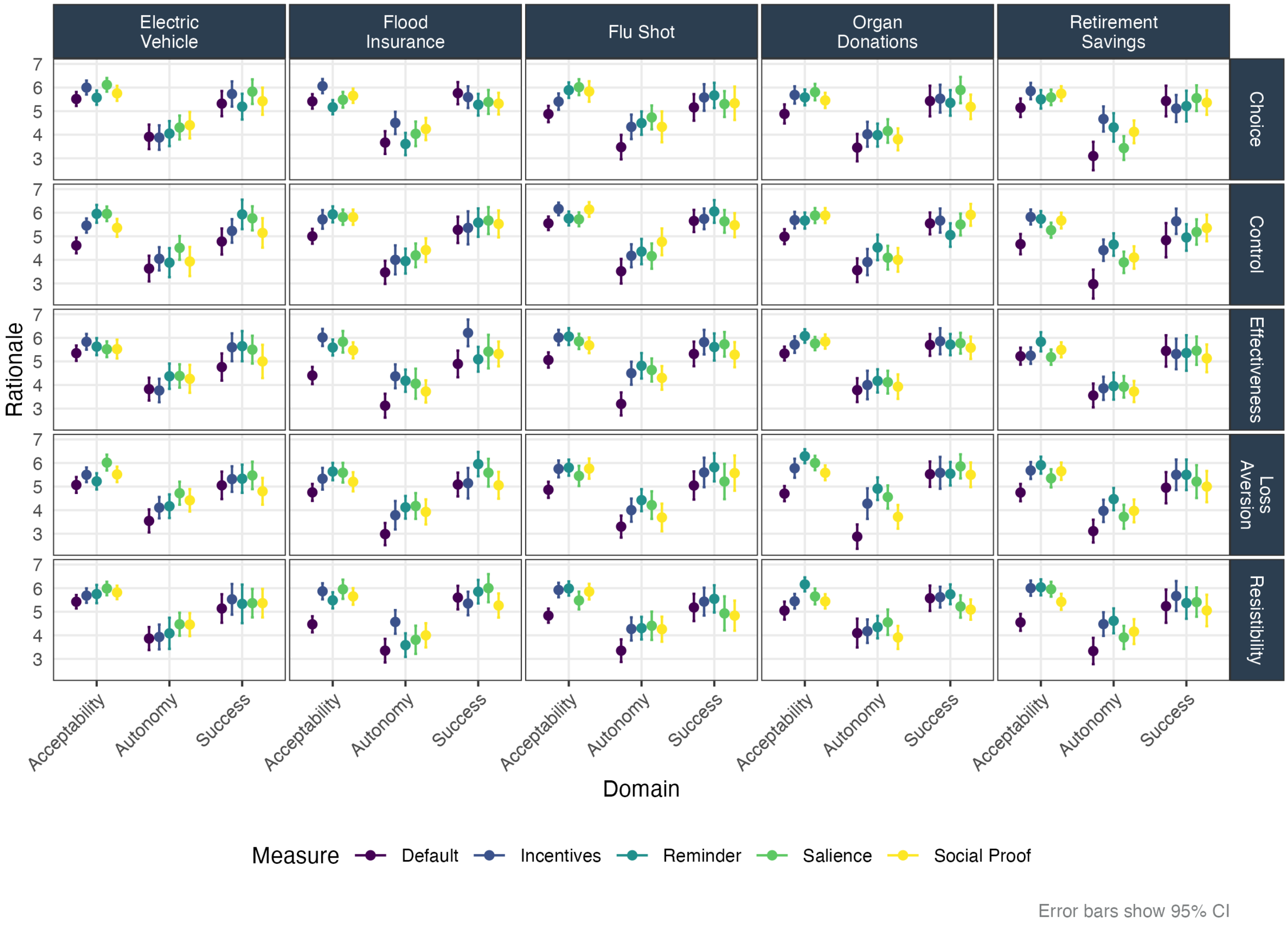

2.3 Exploring differences between levels of Domain

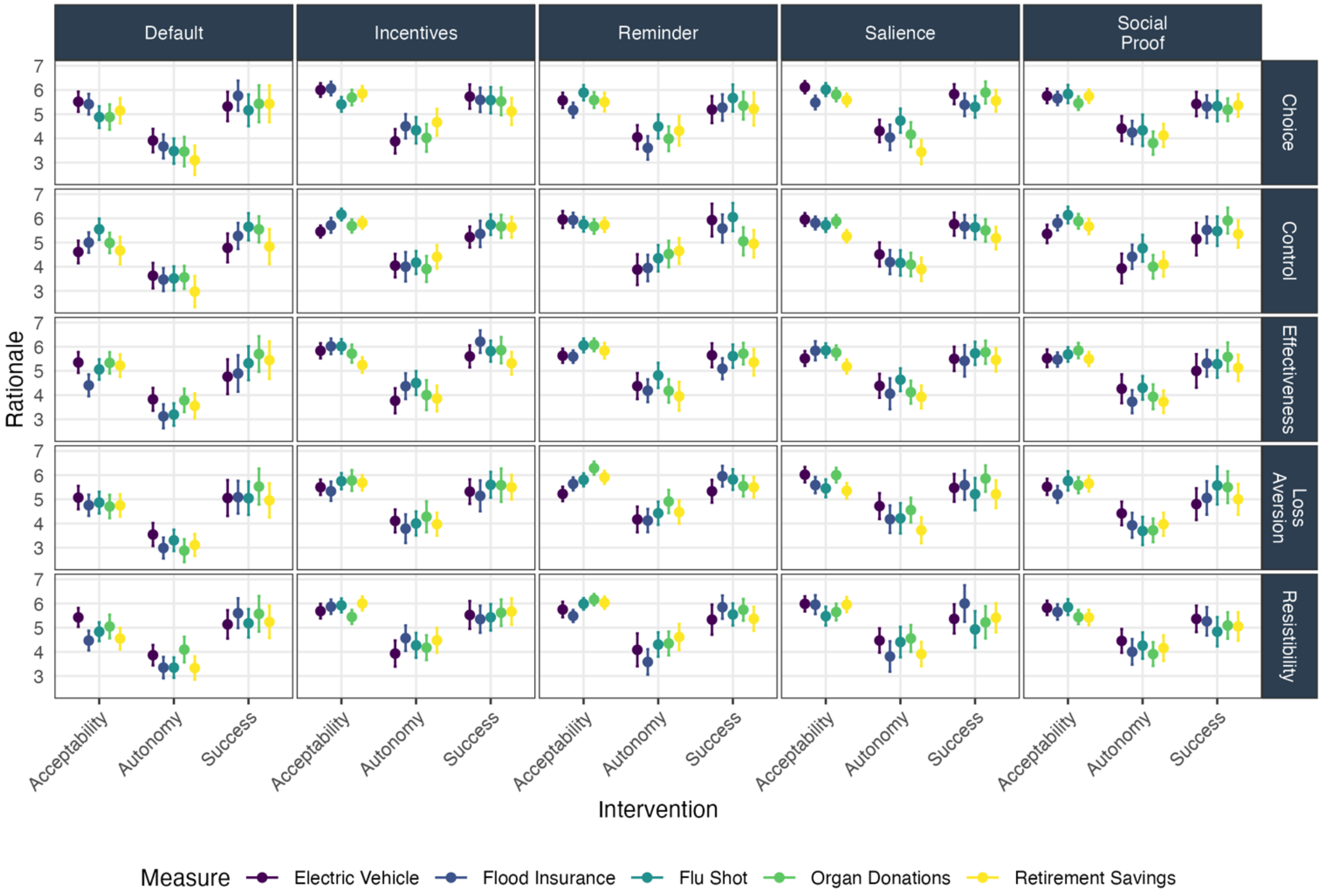

Error bars show 95% CI
