# Supplementary material for: An experimental investigation into whether choice architecture interventions are considered ethical
Source: Sci Rep. 2023 Oct 26;13:18334. doi: 10.1038/s41598-023-44604-7 (PMC10603073; doi:10.1038/s41598-023-44604-7)

An Experimental Investigation Into Whether Choice Architecture Interventions Are Considered Ethical.

Supplementary Information

Daniella Turetski*, Renante Rondina, Jordan Hutchings, Bing Feng, Dilip Soman

Rotman School of Management, University of Toronto, Toronto, ON

***Corresponding authors:** Daniella Turetski (dana.turetski@rotman.utoronto.ca)

Supplementary Information 1 (Pilot Experiment)

Methods

Participants

We aimed to recruit 30 participants per counterbalance group. Participants were adults (18+ years) from the United States or Canada who signed up for the study through Amazon's Mechanical Turk and were randomly assigned to one of two groups. Participants were compensated $4US for completing the experiment and submitting a survey code that they received at the end of the study.

Procedure

The experiment was built and hosted in Qualtrics and was compatible with cellular devices and tablets. All participants provided informed written consent and received a written debriefing. The experiment was run online and participants had no contact with researchers before or during the experiment. The experiment was approved by the Research Ethics Board at the University of Toronto.

Prior to the experiment, all participants were informed that they would have to read five scenarios in which an organization or government was attempting to nudge their customers or citizens. Participants were informed that a nudge is a way of presenting choices and information in such a way to encourage people to select the option or behave in such a way that promotes their best interests or general welfare. Participants were also informed that after each scenario, they would be asked to indicate how much they agree or disagree with the nudge, how much it limits their freedom of choice, and how effective they think the nudge would be.

All stimuli were displayed in black text (RGB: 0, 0, 0) against a white background (255, 255, 255). Each scenario was structured in a similar way with three paragraphs. The first paragraph introduced the organization or government, the behaviour they were trying to change,

and the positive impact it was expected to have on the customers or citizens. The second paragraph described the status quo and the changes that were planned to be made. The third paragraph consisted of one short sentence that either reiterated the benefits or effectiveness of the nudge, the risk of not complying with the target behaviour, or the customer or citizen's ability to resist or opt out of the planned changes. At the bottom of each scenario, participants were asked to indicate how much they agreed or disagreed with the following statements:

- I find the proposed changes acceptable.
- The proposed changes do not threaten my autonomy or freedom of choice.
- I believe the proposed changes will successfully produce the intended effects.

Participants were instructed to indicate their response on a 7-point Likert scale (1 = strongly disagree, 4 = neither agree nor disagree, 7 = strongly agree).

Design

We used a five-factor Latin Cube design with three blocking variables, with Domain, Nudge, and Group as the blocking variables, and Treatment as the analytical variable. Each factor had five levels (See Appendix). For Domain, the first paragraph introduced the scenario by describing a government or organization, the behaviour of their citizens or customers that they were trying to change, and the intended positive outcomes of the behaviour change (Organ Donation, Retirement Savings, Flu Shots, Flood Insurance, Electric Vehicles). For Nudge, the second paragraph described the status quo and the intervention (Defaults, Incentives, Salience, Reminders, Social Proof). For Treatment, the third paragraph contained a short sentence to test different ways that a participant's ratings could be influenced (Control, Effectiveness, Choice, Loss Aversion, Resistibility). In the control condition, no rationale of the intervention was provided. Rather, the intended behaviour change was reiterated. Scenarios were constructed such

that each level of each variable was randomly assigned to each level of the other two variables exactly once, with no pairing occurring more than once. Five versions of the second and third paragraphs were created for each level of Nudge and Treatment so that they would be compatible with the level of Domain that they were assigned to. This produced 25 unique scenarios that were randomly and evenly assigned to one of five Groups such that each level of each factor appeared once in each Group.

Analysis

Preliminary analysis were conducted using the lmerTest ([ref]) and multcomp ([ref]) packages in R (R Development Core Team, 2016). Ratings of acceptability, autonomy, and effectiveness were each analyzed with a type 3 ANOVA with a Satterthwaite correction on a linear mixed effect model with Domain, Nudge, Treatment, and Group as fixed factors, and Subject as a random factor, and no interactions.

Secondary analysis was also conducted using the ordinal (Christensen, 2019) and RVAideMemoire (Herve, 2022) packages. Ratings of acceptability, autonomy, and success were each analyzed with a type 2 analysis of deviance (ANODE) on a cumulative link mixed model with Domain, Nudge, Treatment, and Group as fixed factors, and Subject as a random factor, and no interactions.

Results

One subject from Group 3 was removed from all analyses due to missing data.

A linear mixed effect model on acceptability ratings revealed a main effect of Domain ($F_{(4,583.02)}$ = 3.15, p = 0.0140, partial eta-squared = 0.02), a main effect of Nudge ($F_{(4,583.02)}$ = 10.78, p < 0.0001, partial eta-squared = 0.07), and a main effect of Treatment ($F_{(4,583.02)}$ = 5.93, p = 0.0001, partial eta-squared = 0.04). A cumulative link mixed model on acceptability ratings

also revealed a main effect of Domain (likelihood ratio $X^2_{(4)} = 11.06$, $p = 0.0259$), Nudge (likelihood ratio $X^2_{(4)} = 36.44$, $p < 0.0001$), and Treatment (likelihood ratio $X^2_{(4)} = 20.89$, $p = 0.0003$). Detailed results are reported in Table S1.1 and Figure S1.1.

A linear mixed effect model on autonomy ratings revealed a main effect of Domain ($F_{(4,583.18)} = 2.52$, $p = 0.0402$, partial eta-squared = 0.02), a main effect of Nudge ($F_{(4,583.18)} = 11.35$, $p < 0.0001$, partial eta-squared = 0.07), a main effect of Treatment ($F_{(4,583.18)} = 2.67$, $p < 0.05$, partial eta-squared = 0.02), and a main effect of Group ($F_{(4,583.18)} = 2.45$, $p = 0.031$, partial eta-squared = 0.06). A cumulative link mixed model on autonomy ratings revealed a main effect of Domain (likelihood ratio $X^2_{(4)} = 10.07$, $p = 0.0392$), Nudge (likelihood ratio $X^2_{(4)} = 40.82$, $p < 0.0001$), and Treatment (likelihood ratio $X^2_{(4)} = 9.39$, $p = 0.0389$). Detailed results are reported in Table S1.2 and Figure S1.2.

A linear mixed effect model on success ratings revealed a main effect of Domain ($F_{(4,583.23)} = 3.10$, $p = 0.0152$, partial eta-squared = 0.02), and a main effect of Nudge ($F_{(4,583.23)} = 5.49$, $p = 0.0002$, partial eta-squared = 0.04). A cumulative link mixed model on success ratings revealed a main effect of Nudge (likelihood ratio $X^2_{(4)} = 19.13$, $p = 0.0007$). Detailed results are reported in Table S1.3 and Figure S1.3.

Table S1.1. Coefficient estimates and odds ratios for acceptability ratings.

		Linear Mixed Effect Model					Cumulative Link Mixed Model			
**Factor**	**Category**	**Estimate**	**95% CI**	**df**	**t**	**p**	**OR**	**95% CI**	**z**	**p**
Domain	Electric Vehicle	0.00	-0.23 - 0.23	582.93	0.01	0.9892	0.92	0.92 - 0.93	-28.10	p<0.0001
	Flood Insurance	0.30	0.07 - 0.52	582.93	2.55	0.0111	1.57	1.57 - 1.58	156.56	p<0.0001
	Flu Shot	0.29	0.06 - 0.51	583.17	2.45	0.0146	1.63	1.62 - 1.64	168.52	p<0.0001
	Organ Donation	0.19	-0.04 - 0.41	582.93	1.59	0.1119	1.49	1.48 - 1.50	149.77	p<0.0001
Nudge	Incentives	0.52	0.29 - 0.74	582.93	4.42	p<0.0001	2.54	2.53 - 2.56	321.57	p<0.0001
	Reminder	0.69	0.46 - 0.92	582.93	5.91	p<0.0001	3.56	3.54 - 3.58	452.26	p<0.0001
	Salience	0.59	0.36 - 0.82	583.16	5.06	p<0.0001	2.78	2.77 - 2.80	352.90	p<0.0001
	Social Proof	0.34	0.11 - 0.57	582.93	2.93	0.0036	1.69	1.68 - 1.70	183.32	p<0.0001
Treatment	Choice	0.09	-0.14 - 0.31	582.93	0.75	0.4557	1.21	1.21 - 1.22	66.92	p<0.0001
	Effectiveness	0.04	-0.19 - 0.27	583.17	0.34	0.7324	1.08	1.07 - 1.08	24.99	p<0.0001
	Loss Aversion	0.35	0.12 - 0.58	582.93	2.99	0.0029	1.95	1.94 - 1.96	237.25	p<0.0001
	Resistibility	0.45	0.22 - 0.68	582.93	3.85	0.0001	2.30	2.29 - 2.31	292.38	p<0.0001
Group	2	-0.04	-0.53 - 0.46	143.84	-0.14	0.8901	1.09	0.48 - 2.45	0.21	0.8369
	3	0.02	-0.49 - 0.52	144.06	0.06	0.9506	1.62	1.61 - 1.63	181.86	p<0.0001
	4	-0.18	-0.69 - 0.33	143.84	-0.69	0.4904	0.84	0.35 - 2.02	-0.38	0.7037
	5	-0.45	-0.96 - 0.06	143.84	-1.71	0.0888	0.59	0.25 - 1.38	-1.22	0.2215

Table S1.2. Coefficient estimates and odds ratios for autonomy ratings.

		Linear Mixed Effect Model					Cumulative Link Mixed Model			
**Factor**	**Category**	**Estimate**	**95% CI**	**df**	**t**	**p**	**OR**	**95% CI**	**z**	**p**
Domain	Electric Vehicle	0.25	-0.02 - 0.52	583.07	1.76	0.0782	1.54	0.99 - 2.39	1.92	0.0548
	Flood Insurance	0.41	0.14 - 0.68	583.07	2.95	0.0033	1.89	1.22 - 2.93	2.85	0.0044
	Flu Shot	0.29	0.02 - 0.56	583.36	2.08	0.0380	1.60	1.03 - 2.47	2.10	0.0355
	Organ Donation	0.13	-0.14 - 0.40	583.07	0.97	0.3338	1.21	0.78 - 1.87	0.85	0.3954
Nudge	Incentives	0.72	0.45 - 0.99	583.07	5.19	p<0.0001	3.31	2.11 - 5.21	5.20	p<0.0001
	Reminder	0.76	0.49 - 1.03	583.07	5.46	p<0.0001	2.95	1.90 - 4.59	4.80	p<0.0001
	Salience	0.80	0.53 - 1.07	583.35	5.77	p<0.0001	3.55	2.26 - 5.55	5.53	p<0.0001
	Social Proof	0.53	0.26 - 0.80	583.07	3.80	0.0002	2.35	1.51 - 3.65	3.78	0.0002
Treatment	Control	0.07	-0.20 - 0.34	583.36	0.52	0.6005	1.17	0.75 - 1.81	0.69	0.4926
	Choice	0.07	-0.20 - 0.34	583.34	0.51	0.6114	1.30	0.84 - 2.02	1.20	0.2319
	Loss Aversion	0.27	0.00 - 0.54	583.34	1.96	0.0510	1.59	1.03 - 2.46	2.10	0.0357
	Resistibility	0.38	0.11 - 0.65	583.36	2.75	0.0062	1.89	1.22 - 2.93	2.83	0.0046
Group	2	-0.66	-1.19 - -0.14	143.95	-2.46	0.0152	0.30	0.12 - 0.80	-2.41	0.0161
	3	-0.21	-0.74 - 0.33	144.23	-0.74	0.4576	0.80	0.30 - 2.16	-0.44	0.6587
	4	-0.26	-0.81 - 0.28	143.95	-0.94	0.3497	0.65	0.24 - 1.79	-0.83	0.4087
	5	-0.70	-1.24 - -0.16	143.95	-2.50	0.0135	0.31	0.11 - 0.85	-2.28	0.0227

Table S1.3. Coefficient estimates and odds ratios for success ratings.

		Linear Mixed Effect Model					Cumulative Link Mixed Model			
Factor	Category	Estimate	95% CI	df	t	p	OR	95% CI	z	p
Domain	Retirement Savings	0.10	-0.14 - 0.34	583.12	0.82	0.4140	1.13	0.73 - 1.74	0.56	0.5781
	Flood Insurance	0.29	0.05 - 0.53	583.12	2.35	0.0190	1.44	0.94 - 2.21	1.66	0.0962
	Flu Shot	0.33	0.08 - 0.57	583.40	2.63	0.0089	1.63	1.06 - 2.52	2.21	0.0271
	Organ Donation	0.35	0.11 - 0.59	583.12	2.82	0.0049	1.66	1.08 - 2.56	2.31	0.0211
Nudge	Default	0.15	-0.09 - 0.39	583.12	1.21	0.2275	1.37	0.89 - 2.11	1.41	0.1589
	Incentives	0.52	0.28 - 0.76	583.12	4.22	p<0.0001	2.50	1.61 - 3.88	4.10	p<0.0001
	Reminder	0.40	0.16 - 0.64	583.12	3.24	0.0013	1.89	1.23 - 2.91	2.91	0.0037
	Salience	0.28	0.04 - 0.52	583.40	2.23	0.0262	1.54	1.01 - 2.36	2.00	0.0459
Treatment	Effectiveness	0.11	-0.13 - 0.35	583.41	0.86	0.3875	1.19	0.77 - 1.85	0.79	0.4295
	Choice	0.01	-0.23 - 0.25	583.12	0.07	0.9468	1.01	0.66 - 1.57	0.06	0.9493
	Loss Aversion	0.03	-0.22 - 0.27	583.12	0.20	0.8392	0.97	0.63 - 1.49	-0.13	0.8975
	Resistibility	0.09	-0.15 - 0.33	583.12	0.73	0.4673	1.06	0.69 - 1.63	0.27	0.7877
Group	2	-0.28	-0.73 - 0.18	143.99	-1.17	0.2429	0.55	0.22 - 1.38	-1.28	0.2022
	3	-0.23	-0.70 - 0.23	144.28	-0.97	0.3362	0.70	0.27 - 1.81	-0.73	0.4665
	4	-0.44	-0.92 - 0.03	143.99	-1.80	0.0744	0.44	0.17 - 1.16	-1.66	0.0976
	5	-0.59	-1.06 - -0.12	143.99	-2.44	0.0157	0.35	0.14 - 0.91	-2.16	0.0310

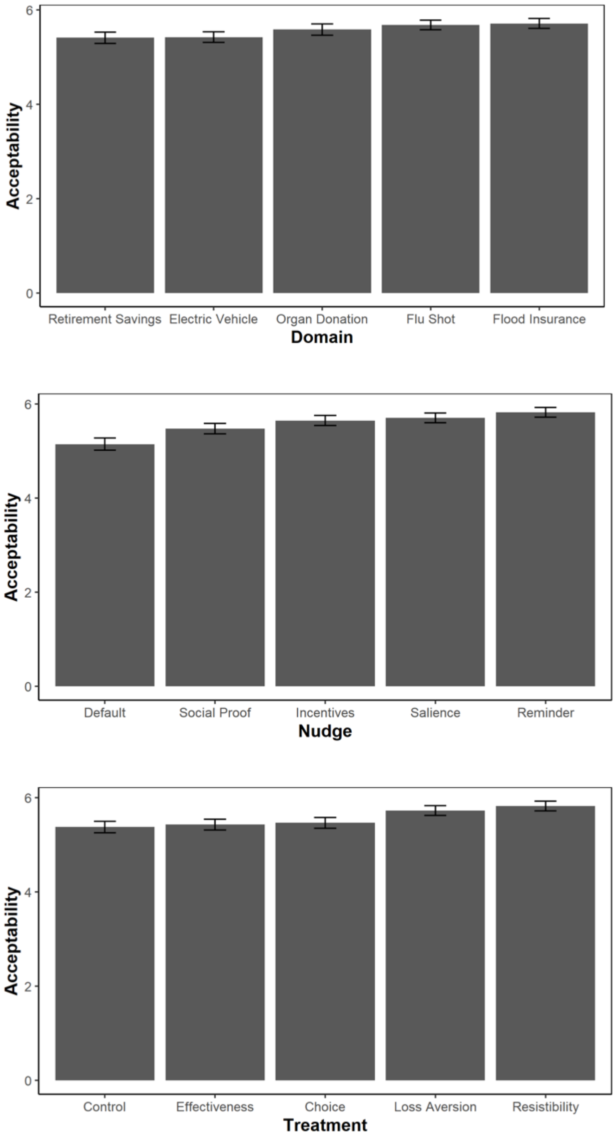

Figure S1.1. Ratings of acceptability. (A) Retirement Savings are less acceptable than Flu Shots and Flood Insurance. (B) Defaults are less acceptable than all other nudges. (C) Control is less acceptable than Loss Aversion and Resistibility. Bars are +/- standard errors.

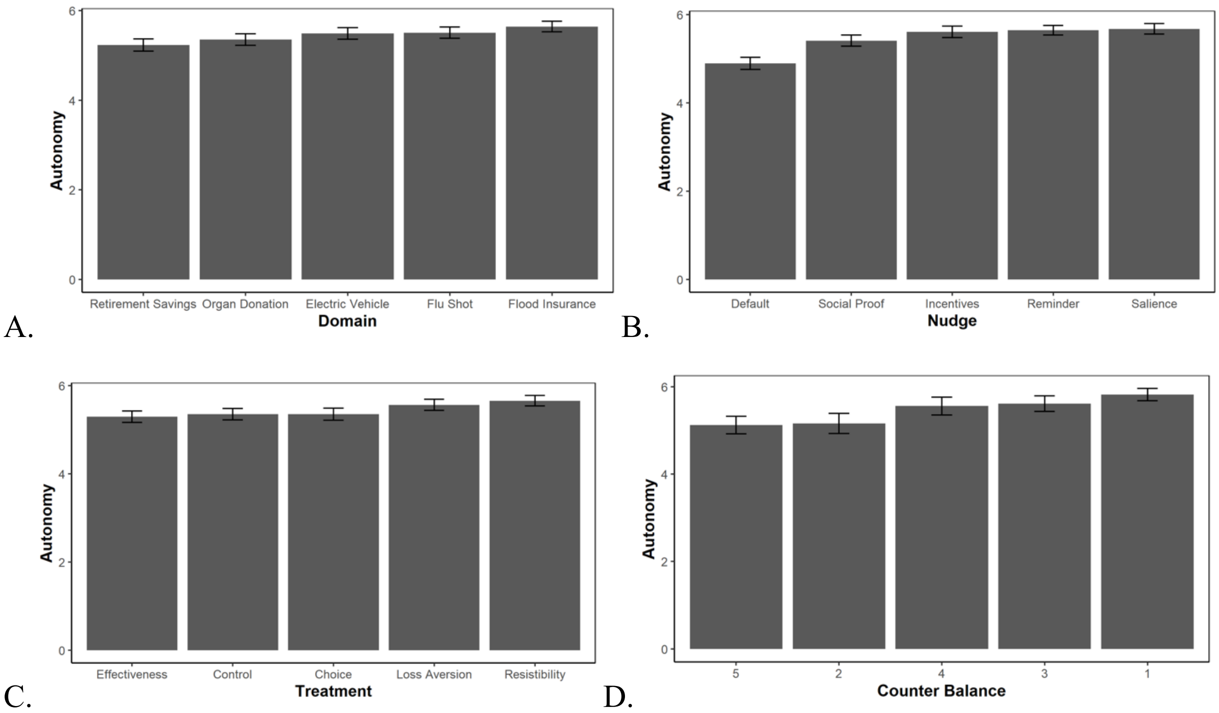

Figure S1.2. Ratings of autonomy. (A) Retirement Savings are more threatening to autonomy than Flu Shots and Flood Insurance. (B) Defaults are more threatening to autonomy than all other nudges. (C) Effectiveness is more threatening to autonomy than Resistibility. (D) Group 1 reported feeling less threat to autonomy than Group 2 and Group 5. Bars are +/- standard errors.

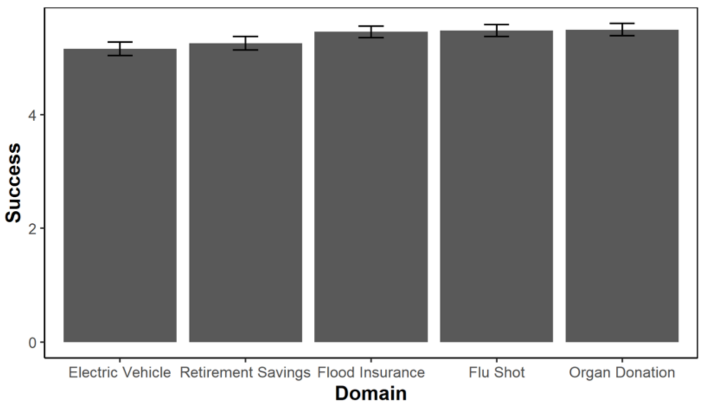

A.

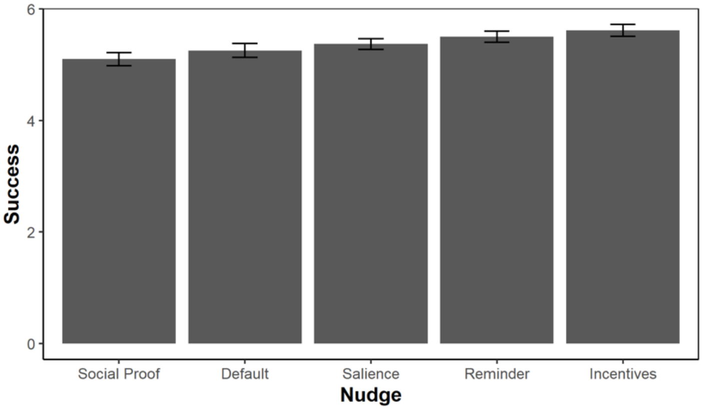

B.

Figure S1.3. Ratings of success. (A) Electric Vehicles are less successful than Flood Insurance, Flu Shots, and Organ Donations. (B) Social Proof are less successful than Salience, reminders, and Incentives. Bars are +/- standard errors.

Supplementary Information 2 (Scenarios for the Registered Study)

	Organ Donation	Flood Insurance	Flu Shot
**Paragraph 1**	The government wants to encourage its citizens to participate in an organ donor program in which they can agree to donate their organs after they die. This is important because more organs will be available to transplant patients when they need them. You receive a letter from the government informing you of their efforts and that you can choose to become an organ donor when you renew your driver's license.	An insurance company wants to encourage its existing policyholders to purchase flood insurance. This is important for people who live in areas with high risk of flooding so that they can be covered for property damage in the event of a flood. You receive a letter from the insurance company informing you of their efforts and that you can choose to purchase flood insurance when you renew your home insurance.	A medical clinic franchise wants to encourage their patients to get the seasonal flu shot. This is important because it can reduce the risk of serious illness. You receive a letter from the clinic franchise informing you of their efforts and that you can choose to get a flu shot whenever you come to a clinic for an appointment.
**Paragraph 2**			
**Default**	Currently, you must actively choose to be an organ donor such that, if you do not make a choice, it is assumed that you do not want to be an organ donor. Under the new changes, it would be assumed that you agree to be an organ donor such that you must actively decline if you do not wish to do so.	Currently, you must actively choose to purchase flood insurance such that, if you do not make a choice, it is assumed that you do not want flood insurance. Under the new changes, it would be assumed that you want to purchase flood insurance such that you must actively decline if you do not wish to do so.	Currently, you must actively ask the staff or physician for a flu shot such that, if you do not ask for it, it is assumed that you do not want to get the flu shot. Under the new changes, the physician would assume that you agree to get a flu shot **(barring any allergies or medical restrictions)** such that you must actively decline if you do not wish to do so.
**Social Proof**	Currently, the letter describing the organ donor program does not provide any information on the number of people that have already agreed to participate in the program. Under the new changes, information will be provided that many people have already agreed to participate.	Currently, the letter describing the flood insurance does not provide any information on the number of people in your area that have already purchased it. Under the new changes, information would be provided that many people have already purchased it.	Currently, the letter describing the flu shot does not provide any information on the number of people who have already received it. Under the new changes, information would be provided that many people have already received it.
**Incentives**	Currently, there are no financial incentives to participate in the organ donor program. Under the new changes, you would receive a small tax rebate for agreeing to participate in the program.	Currently, there are no financial incentives to purchase flood insurance. Under the new changes, you would receive **a discounted rate on your home insurance** if you purchased the flood insurance.	Currently, there are no financial incentives to get a flu shot. Under the new changes, you would receive a discount at a partner pharmacy if you received your shot.
**Salience**	Currently, you are asked if you want to be an organ donor at the bottom of one of the pages of your driver's license renewal form. Under the new changes, you would be asked to be an organ donor in large bold font at the top of the first page of the renewal form.	Currently, you are asked if you want to purchase flood insurance at the bottom of one of the pages of your home insurance renewal form. Under the new changes, you would be asked to purchase flood insurance in large bold font at the top of the first page of the form.	Currently, you are asked if you want to receive the flu shot at the end of your appointment. Under the new changes, you would be asked to receive the flu shot while you are waiting in the exam room.
**Reminder**	Currently, you would only be notified of the option to participate in the program when you are renewing your driver's license. Under the new changes, you would be notified of the option to participate when you receive a reminder to renew your driver's license.	Currently, you would only be notified of the option to purchase flood insurance when you are renewing your home insurance. Under the new changes, you would be notified of the option to purchase flood insurance when you receive a reminder to renew your home insurance.	Currently, you would only be notified of the option to receive a flu shot when you come in for your appointment. Under the new changes, you would be notified of the option to receive the flu shot when you receive a reminder for your appointment.

Paragraph 3			
**Control**	The government hopes that this change will help encourage people to participate in the organ donor program.	The company hopes that this change will help encourage people to purchase flood insurance.	The clinic hopes that this change will encourage people to get the flu shot.
**Loss Aversion**	The government explains that this change is designed to prevent the loss of lives.	The company explains that this change is designed to help prevent financial loss from property damage.	The clinic explains that this change is designed to help prevent you from getting sick.
**Resistibility**	The government explains that if you do not want to be an organ donor, then you can still select the option to not participate in the program.	The company explains that if you do not want flood insurance, then you can still select the option to not purchase it.	The clinic explains that if you do not want a flu shot, then you can still select the option to not get one.
**Choice**	The government explains that you can opt out of this change so that you will never have to experience it whenever you renew your license.	The company explains that you can opt out of this change so that you will never have to experience it whenever you renew your home insurance.	The clinic explains that you can opt out of this change so that you will never have to experience it whenever you come to the clinic.
**Effectiveness**	The government explains that, based on research, this change will make people more likely to participate in the organ donor program.	The company explains that, based on research, this change will make people more likely to purchase flood insurance.	The clinic explains that, based on research, this change will make people more likely to get the flu shot.

	Retirement Savings	Electric Vehicles	Baseline Condition[1]
**Paragraph 1**	The government wants to encourage its citizens to deposit their tax refund into their retirement savings account. This is important because it can help people prepare for retirement. You receive a letter from the government informing you of their efforts and that you can contribute to your retirement savings account when you receive your notice of assessment.	A car buying website wants to encourage its users to purchase electric vehicles. This is important because it helps protect the environment while saving owners money on gas and maintenance. You receive an e-mail from the website informing you of their efforts and that you can choose an electric vehicle when you purchase a car through their website.	The government wants to encourage its citizens to complete census reports. This is important because tracking census information helps the government understand the demographics of their nation. You receive a letter from the government informing you of their efforts and that you can complete the census report and mail it back.
**Paragraph 2 (Type)**			
**Default**	Currently, you must actively choose to transfer your tax refund to your savings account such that, if you do not arrange the transfer, it is assumed that you do not want to do so. Under the new changes, it would be assumed that you agree to deposit your refund into your savings account such that you must actively decline if you do not wish to do so.	Currently, you must actively set your search criteria to **filter/sort** by fuel type, if you do not it is assumed that you do not have a preference. Under the new changes, it would be assumed that you have a preference for electric vehicles such that you must actively set your search criteria to not **filter/sort** by fuel type.	Currently, you must actively select a religion when completing the census report. If you do not complete this section, there is no religion data entered on behalf of you. Under the new changes, it would be assumed that you are Christian unless you state otherwise in your census report.

[1] This scenario was adapted from Sunstein (2016)'s list of unpopular nudges (it ranked as the second most unpopular nudge).

**Social Proof**	Currently, the letter describing the tax refund does not provide any information on the number of people who have already deposited it into their savings account. Under the new changes, information would be provided that many people have already deposited it into their savings account.	Currently, the email describing the electric vehicles does not provide any information on the number of people who already own an electric vehicle. Under the new changes, information would be provided that many people already own electric vehicles.	
**Incentives**	Currently, there are no financial incentives to deposit your tax refund into a savings account. Under the new changes, you would receive a special interest rate if you deposited your refund into a savings account.	Currently, there are no financial incentives to purchase an electronic vehicle. Under the new changes, you would receive **a discount on the listed price** if you choose to purchase an electric vehicle.	
**Salience**	Currently, you are asked if you want to deposit your tax refund into a savings account at the bottom of your notice of assessment. Under the new changes, you would be asked to deposit your tax refund into a savings account in large bold font at the top of your notice of assessment.	Currently, you must read through a vehicle's description to see if it is an electric vehicle. Under the new changes, each electric vehicle will be listed with a green leaf logo on the **results page** of your search.	
**Reminder**	Currently, you would only be notified of the option to deposit your tax refund into a savings account when you receive your notice of assessment. Under the new changes, you would be notified of the option to deposit your tax refund into a savings account when you are filing your taxes.	Currently, you would only be notified of the option to purchase an electric vehicle when you are setting your search criteria. Under the new changes, you would be notified of the option to purchase an electric vehicle by a large clickable banner above the **search bar**.	
**Paragraph 3 (Rationale)**			
**Control**	The government hopes that this change will encourage people to deposit their tax refund into a retirement savings account.	The car buying website hopes that this change will encourage people to purchase an electric vehicle.	The government hopes that this change will encourage people to complete their census reports.
**Loss Aversion**	The government explains that this change is designed to help you **avoid financial problems in the future**.	The car buying website explains that this change is designed to **prevent global warming and help you spend less money**.	
**Resistibility**	The government explains that if you do not want to deposit your tax refund into your retirement savings account, then you can still select the option to not do so.	The car buying website explains that if you do not want to see their electric vehicles, then you can still select the option to not do so.	
**Choice**	The government explains that you can opt out of this change so that you will never have to experience it whenever you get a tax refund.	The car buying website explains that you can opt out of this change so that you will never have to experience it whenever you purchase a car.	
**Effectiveness**	The government explains that, based on research, this change will make people more likely to deposit their tax refund into a retirement savings account.	The car buying website explains that, based on research, this change will make people more likely to purchase an electric vehicle.	

Supplementary Information 3 (Original Design Table from the Registration)

Question	Hypothesis	Sampling plan	Analysis Plan	Interpretation given to different outcomes
Does the acceptability of an intervention depend on the type of intervention?	Acceptability ratings will vary depending on the type of intervention used.	550 adults (18+ years) from the United States or Canada who sign up for the study through Amazon's Mechanical Turk, pass our attention checks (two quality assurance questions and a non-sensical question attention check), and complete all of the study's questions.	We will average ratings for the three statements measuring acceptability to create an acceptability score. Acceptability scores will be fitted into a cumulative link mixed model with Domain, Intervention, and Rationale as fixed factors and Subject as a random factor and analyzed with a type 3 ANOVA. As exploratory analyses, we will redo this with all possible interactions, and we will run post hoc analyses explore differences between specific levels within factors (correcting for multiple comparisons with Bonferroni corrections). We will be using an alpha level of .05 for all our analyses.	The acceptability of an intervention may depend on a combination of different factors including, but not exclusive to, the Domain, Intervention, and Rationale.
Does the acceptability of an intervention depend on the domain in which the intervention is delivered?	Acceptability ratings will vary depending on the domain in which the intervention is implemented.			
Does the acceptability of an intervention depend on the rationale given for it?	Acceptability ratings will vary depending on the rationale used to explain the implementation and benefits.			
Does the perceived threat to autonomy and freedom of choice of an intervention depend on the	Perceived threat to autonomy will vary depending on the type of		We will average ratings for the three statements measuring threat to autonomy to create an autonomy score. Autonomy scores will be fitted into a cumulative link mixed model with Domain, Intervention,	The perceived threat of autonomy and freedom of choice of an intervention may depend on a combination of different factors including, but not exclusive to, the

type of intervention?	intervention used.		and Rationale as fixed factors and Subject as a random factor and analyzed with a type 3 ANOVA. As exploratory analyses, we will redo this with all possible interactions, and we will run post hoc analyses explore differences between specific levels within factors (correcting for multiple comparisons with Bonferroni corrections). We will be using an alpha level of .05 for all our analyses.	Domain, Intervention, and Rationale.
Does the perceived threat to autonomy and freedom of choice of an intervention depend on the domain in which an intervention is delivered?	Perceived threat to autonomy will vary depending on the domain in which the intervention is implemented.			
Does the perceived threat to autonomy and freedom of an intervention choice depend on the rationale given for it?	Perceived threat to autonomy will vary depending on the rationale used to explain the implementation and benefits.			

Supplementary Information 4 (Participant Numbers per Group – Registered Study)

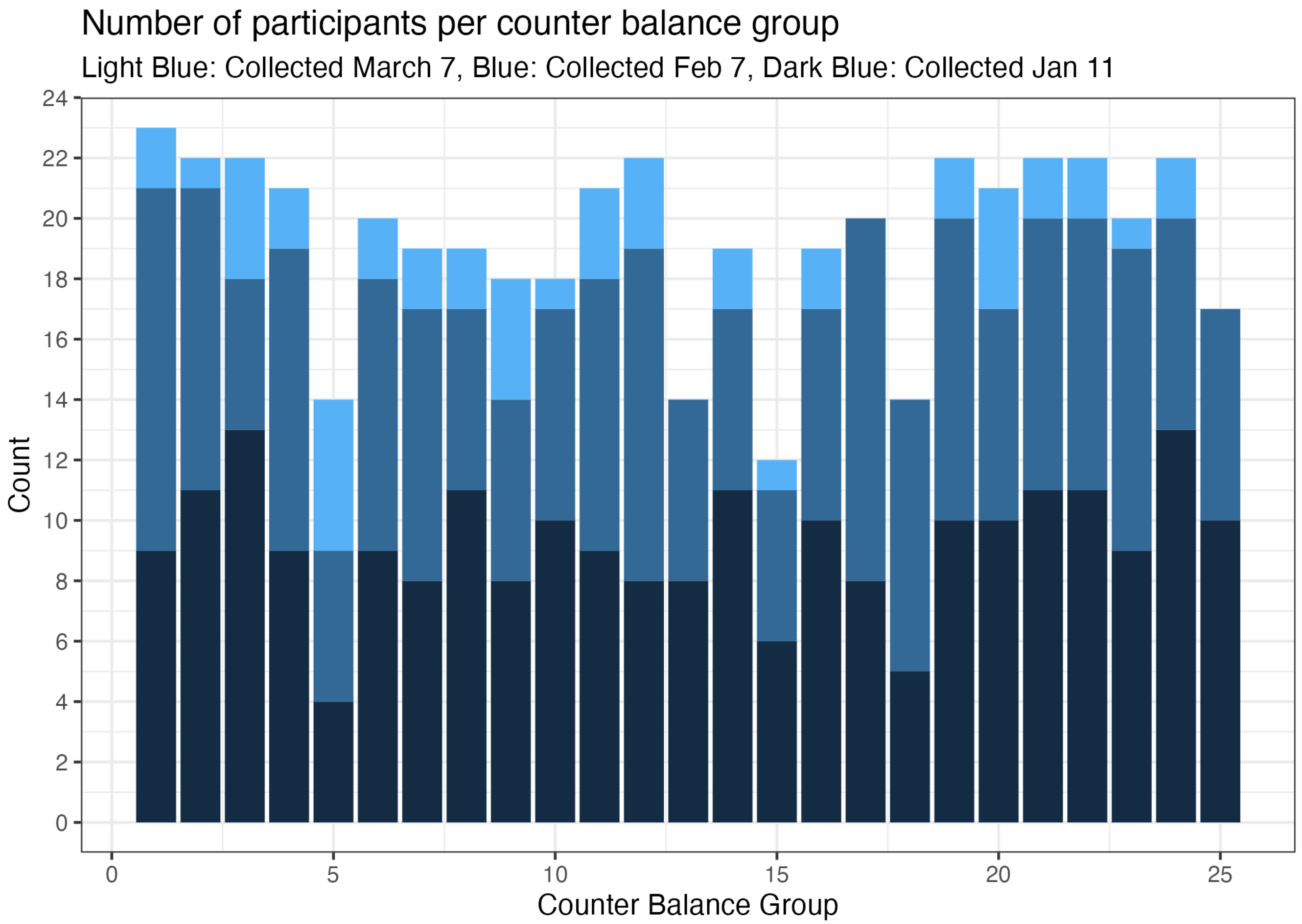

Supplementary Information 5 (Main Analyses Using Initial Batch of Participants)

Table S.5 Analysis of Deviance Across Interaction Models – First batch of participants

	Acceptability				Autonomy				Success			
	ChiSq	Df	P-value		ChiSq	Df	P-value		ChiSq	Df	P-value	
Domain	24.282	4	<0.001	***	9.213	4	0.056	*	4.651	4	0.325	
Intervention	141.161	4	<0.001	***	88.065	4	<0.001	***	25.296	4	<0.001	***
Rationale	7.930	4	0.094	*	2.793	4	0.593		2.766	4	0.598	
Domain*Intervention	44.271	16	<0.001	***	11.326	16	0.789		18.340	16	0.304	
Domain*Rationale	29.973	16	0.018	**	8.690	16	0.926		16.025	16	0.451	
Intervention*Rationale	27.837	16	0.033	**	9.745	16	0.88		30.112	16	0.017	**
Domain*Intervention*Rationale	75.268	64	0.158		42.049	64	0.985		43.582	64	0.976	
Counter Balance	-0.109	24	1		-0.104	24	1		0.022	24	1	

Figure S.5: Average ratings of acceptability and perceived threat to autonomy for Intervention level – First batch of participants

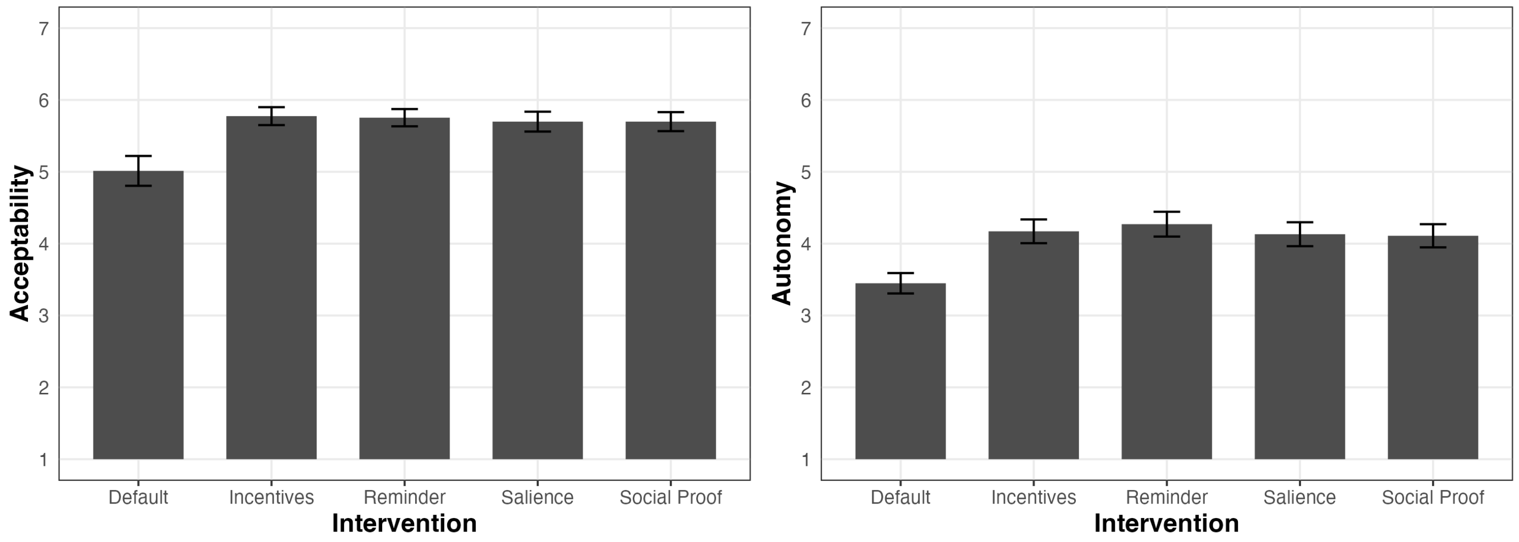

Error bars represent +/- 1 SE.

Supplementary Information 6 (Bonferroni Corrected Plots of Differences Within One Factor Holding Other Two Constant)

Figure S.6.1 Exploring differences between levels of Rationale

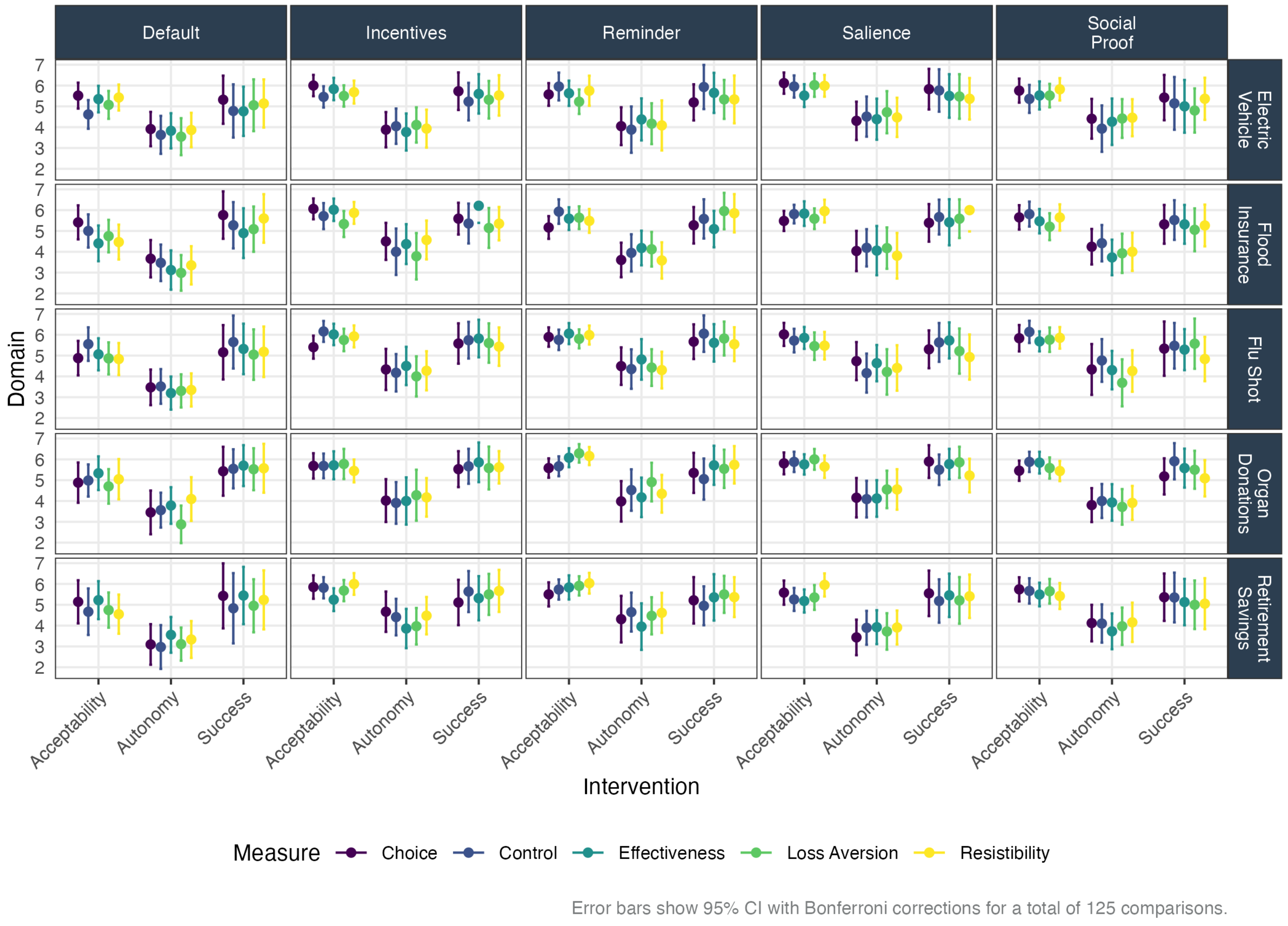

Figure S.6.2 Exploring differences between levels of Intervention

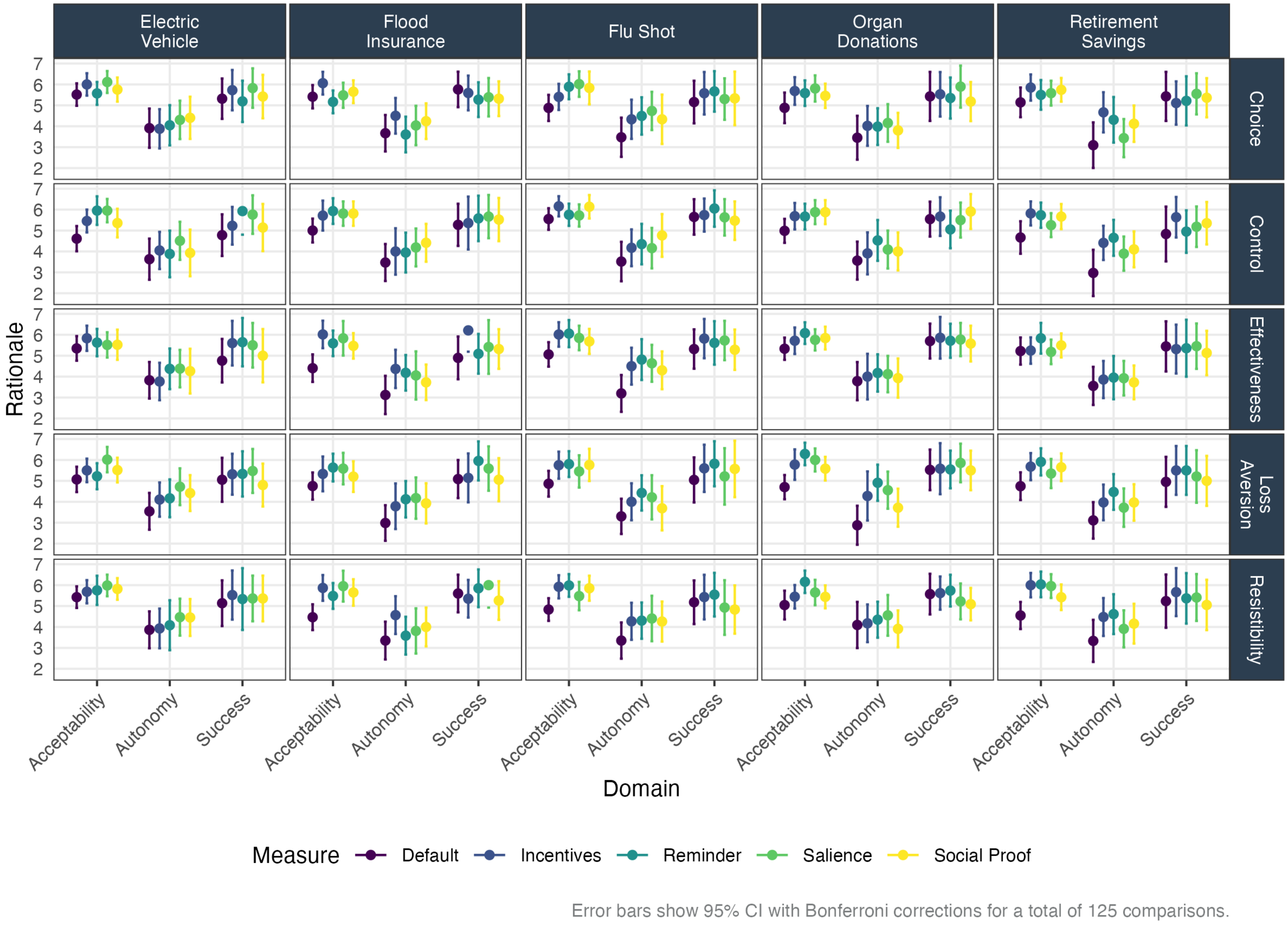

Figure S.6.3 Exploring Differences between Levels of Domain

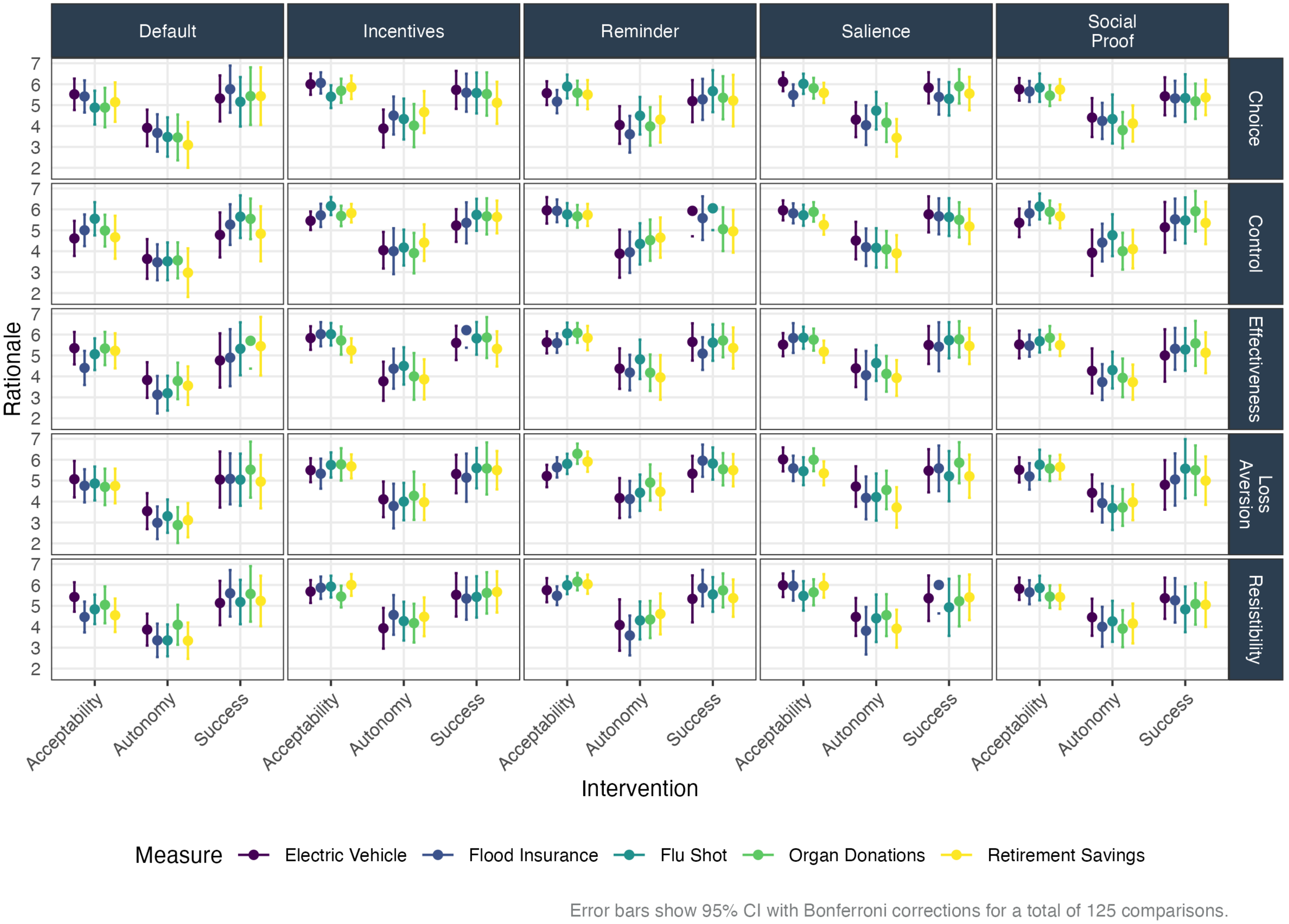

Supplement: Supplementary file 2 — Supplementary Information. [file 41598_2023_44604_MOESM2_ESM.pdf]
