# Supplementary material for: An experimental investigation into whether choice architecture interventions are considered ethical
Source: Sci Rep. 2023 Oct 26;13:18334. doi: 10.1038/s41598-023-44604-7 (PMC10603073; doi:10.1038/s41598-023-44604-7)
Supplement: Supplementary file 3 — Figure Legend. [file 41598_2023_44604_MOESM3_ESM.pdf]

Figure and Table Legend

Table Legend:

Table 1:

Caption: *The main effects of Domain, Intervention, and Rationale, as well as all 2-way and 3-way interactions. Note: We include counterbalance group and batch date as factors to check for any effects of group assignment and date of batch collection.*

Analysis of Deviance Across Interaction Models

	Acceptability				Autonomy				Success			
	ChiSq	Df	P-value		ChiSq	Df	P-value		ChiSq	Df	P-value	
Domain	25.723	4	<0.001	***	23.659	4	<0.001	***	12.782	4	0.012	**
Nudge	283.411	4	<0.001	***	195.679	4	<0.001	***	24.865	4	<0.001	***
Rationale	6.690	4	0.153		3.579	4	0.466		2.035	4	0.729	
Domain*Nudge	33.671	16	0.006	***	15.955	16	0.456		19.241	16	0.256	
Domain*Rationale	23.483	16	0.101		8.166	16	0.944		8.425	16	0.935	
Nudge*Rationale	31.580	16	0.011	**	14.567	16	0.557		10.605	16	0.833	
Domain*Nudge*Rationale	68.694	64	0.321		39.723	64	0.993		40.448	64	0.991	
BatchDate	2.766	2	0.251		11.245	2	0.004	***	1.478	2	0.478	
Counter Balance	-0.116	24	1		-0.225	24	1		-0.006	24	1	

Test statistics from Cumulative Link Mixed Model (CLMM) with interactions between all factors. The test statistic follows a χ^2 distribution, and as a result the ChiSq test statistics are shown with their corresponding p-values. Each set of columns corresponds to the dependent variable used (Acceptability, Autonomy, or Success). The rightmost column of results for each DV indicates the significance of the test statistic; $*** \, p < 0.01$, $** \, p < 0.05$, $* \, p < 0.1$. BatchDate is an indicator for the date of data collection, counter balance is the counter balance group each repondent was assigned to.

Figure Legend:

Figure 1: caption: *average ratings of acceptability and perceived threat to autonomy for each level of Intervention.*

Figure 1: average ratings of acceptability and perceived threat to autonomy for each level of Intervention

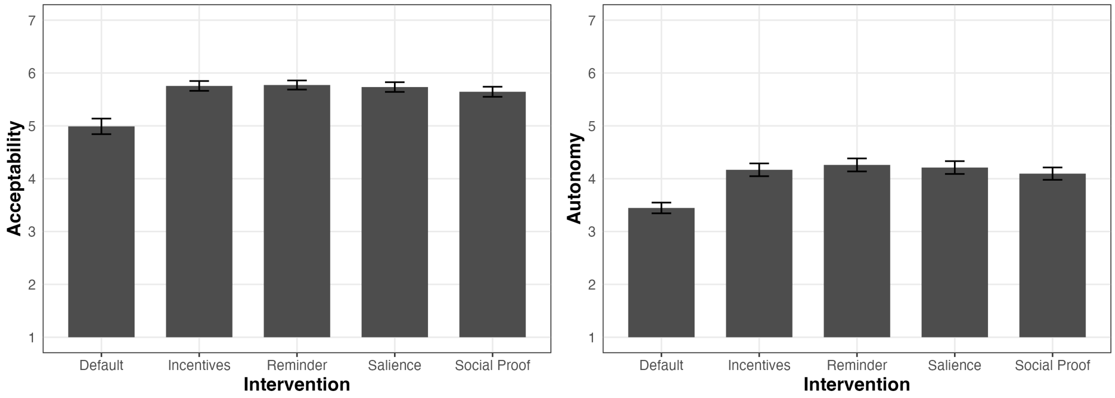

Error bars represent +/- 1 SE.

Average ratings are shown for the Accetpability (left) and Autonomy (right) DVs. Error bars represent the $\pm$ 1 standard error of the mean, computed by the R function Rmisc::summarySE().

Appendix Figure 2: caption: *Baseline Condition Results*

Baseline Condition Responses

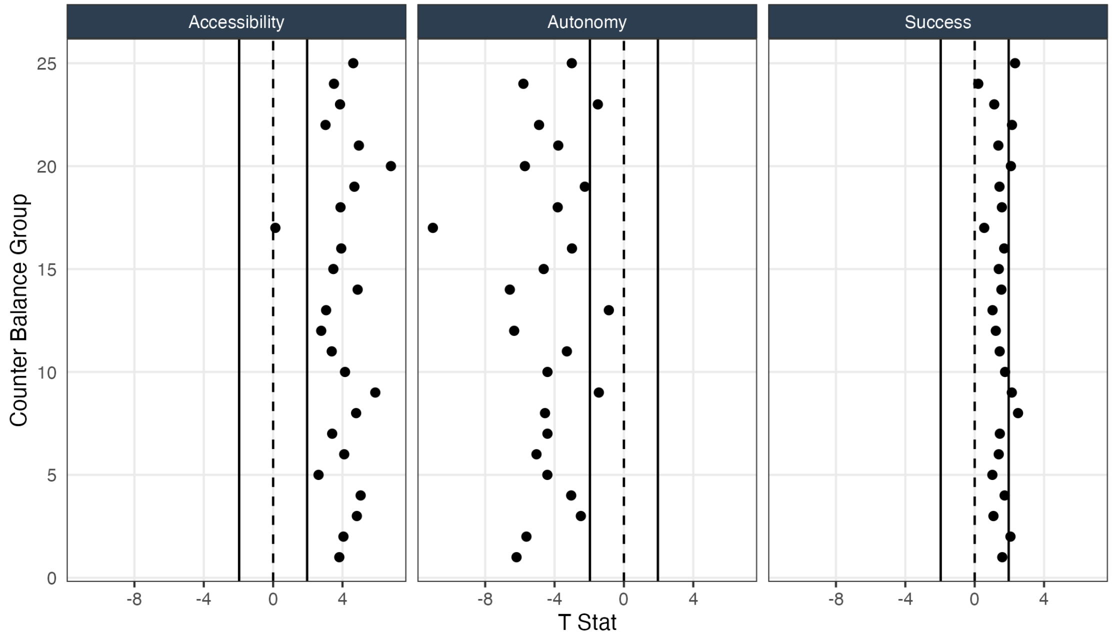

Notes:
Mean responses in the baseline and experiment conditions were compared with two-way paired t-test across all counter balance groups. This plot does not correct for multiple tests, and should be viewed as descriptive evidence that participants responded differently in the main experiment than in the baseline scenario.

Each panel of the figure shows the two-way paired t-test statistic comparing the mean response between response scores provided in the baseline scenario and in the

experiment. A test is preformed at each counter balance group. Solid lines indicate ± 1.96, suggestive of a 95% confidence interval under the hypothesis that the difference between responses in the baseline do not differ from in the experiment.

Appendix Figure 3: caption: *Exploring differences between levels of Domain*

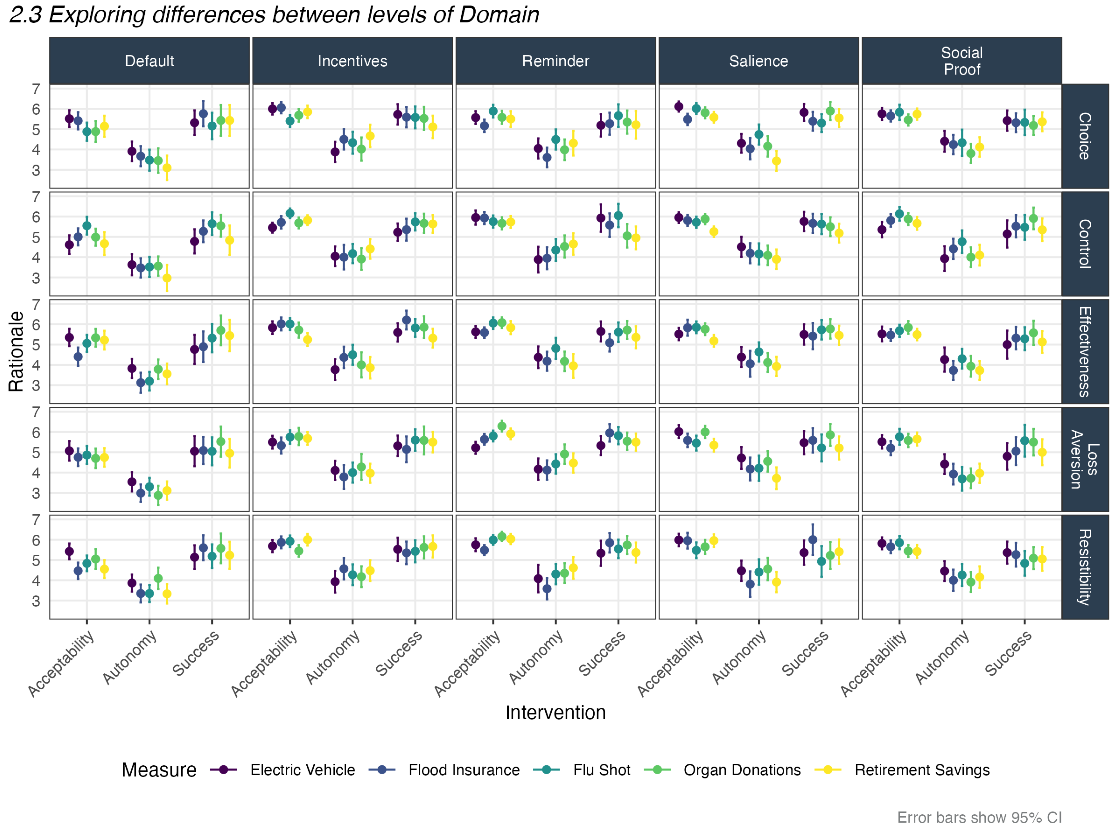

Figure 3 - 5 all share the same interpretation. Each plot varies which of the three factors (Intervention, Rationale, Domain) the responses are averaged over. Each individual panel (of the 25 total) shows the average responses across each level of the omitted factor. Error bands are shown as the mean response ±1.96 × SE. The error bars are meant to provide suggestive evidence of scenarios which a given system of choice architecture may be favourable to another. Each panel is constructed by holding the two levels of the factor fixed. e.g., the top-left most panel holds Intervention as Default, and the Rationale as Choice, then averages over the different Domain responses, showing each average for our Acceptability, Autonomy, and Success DVs.

Appendix Figure 4: caption: *Exploring differences between levels of Intervention*

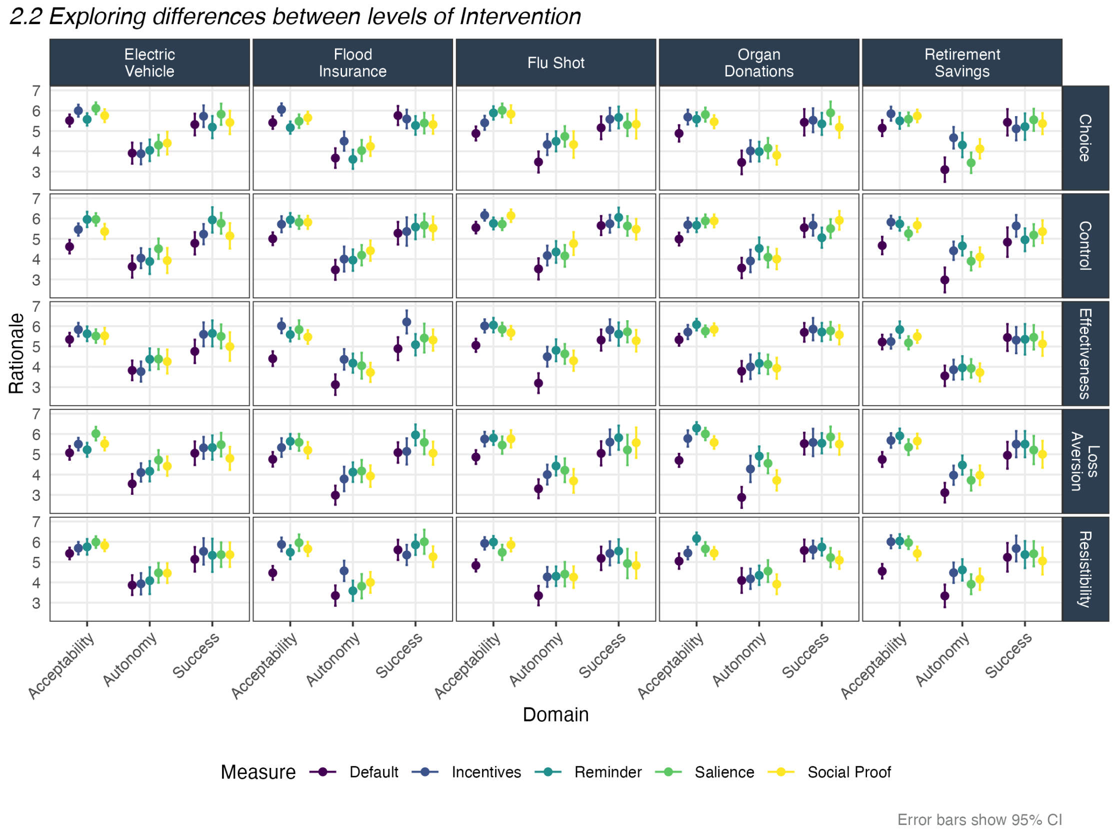

Described in Figure 3, however holding Domain and Rationale constant.

Appendix Figure 5: caption: *Exploring differences between levels of Rationale*

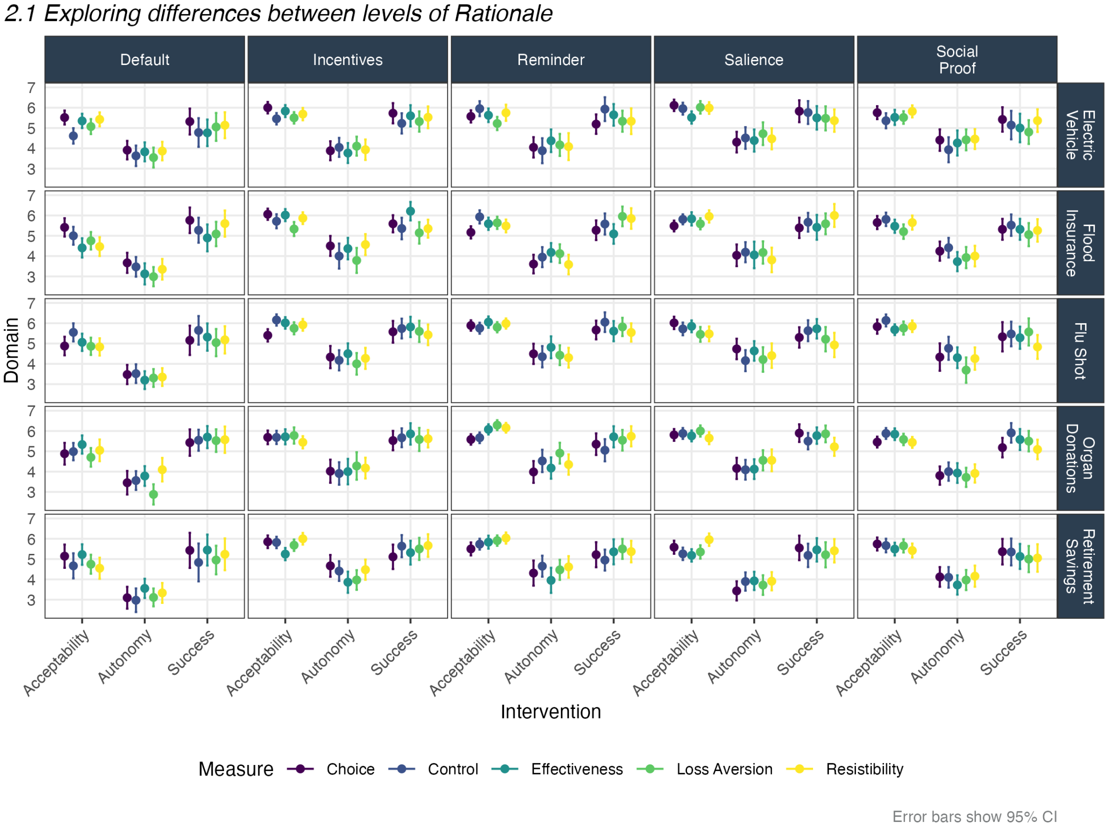

Described in Figure 3, however holding Intervention and Domain constant.
